# Quintet Rooting: rooting species trees under the multi-species coalescent model

**DOI:** 10.1093/bioinformatics/btac224

**Published:** 2022-06-27

**Authors:** Yasamin Tabatabaee, Kowshika Sarker, Tandy Warnow

**Affiliations:** Department of Computer Science, University of Illinois at Urbana-Champaign, Urbana, IL 61801, USA; Department of Computer Science, University of Illinois at Urbana-Champaign, Urbana, IL 61801, USA; Department of Computer Science, University of Illinois at Urbana-Champaign, Urbana, IL 61801, USA

## Abstract

**Motivation:**

Rooted species trees are a basic model with multiple applications throughout biology, including understanding adaptation, biodiversity, phylogeography and co-evolution. Because most species tree estimation methods produce unrooted trees, methods for rooting these trees have been developed. However, most rooting methods either rely on prior biological knowledge or assume that evolution is close to clock-like, which is not usually the case. Furthermore, most prior rooting methods do not account for biological processes that create discordance between gene trees and species trees.

**Results:**

We present Quintet Rooting (QR), a method for rooting species trees based on a proof of identifiability of the rooted species tree under the multi-species coalescent model established by Allman, Degnan and Rhodes (*J. Math. Biol.*, 2011). We show that QR is generally more accurate than other rooting methods, except under extreme levels of gene tree estimation error.

**Availability and implementation:**

Quintet Rooting is available in open source form at https://github.com/ytabatabaee/Quintet-Rooting. The simulated datasets used in this study are from a prior study and are available at https://www.ideals.illinois.edu/handle/2142/55319. The biological dataset used in this study is also from a prior study and is available at http://gigadb.org/dataset/101041.

**Contact:**

warnow@illinois.edu

**Supplementary information:**

Supplementary data are available at *Bioinformatics* online.

## 1 Introduction

Phylogenetic trees provide insight into many biological questions and are typically estimated using statistical methods that assume a model of evolution. While rooted phylogenies are the final objective, estimated gene trees (i.e. trees on a single locus) and estimated species trees (i.e. trees considering multiple loci and potentially full genomes) are usually unrooted: gene trees are generally estimated under time-reversible models of sequence evolution, and species trees are then estimated under models for gene evolution within species trees, using techniques such as ASTRAL (Mirarab *et al.*, 2014a), StarBEAST2 (Ogilvie *et al.*, 2017) or SVDquartets (Chifman and Kubatko, 2014) that also do not produce rooted trees. As a result, additional techniques for rooting species trees are used.

When evolutionary rates follow a strict molecular clock (so that the expected number of substitutions is proportional to time), then rooting trees is straightforward. However, since evolution does not follow the strict molecular clock [see discussion and references in Pascual-García *et al.* (2019)], ‘relaxed clock’ models have been proposed and then used to root phylogenies (Lepage *et al.*, 2007). Outgroup rooting (i.e. rooting the species tree on the edge that separates the outgroup species from the rest of the species) is another common approach. Both relaxed clock and outgroup rooting methods are used, but no methods have been found to be entirely successful (Wertheim *et al.*, 2010; Wilberg, 2015). Moreover, very few methods for rooting species trees have been developed that specifically address genome-scale processes, such as incomplete lineage sorting (ILS) or gene duplication and loss, that result in discordance between gene trees and species trees (Degnan and Rosenberg, 2009; Maddison, 1997).

Here, we introduce a new statistical method, ‘Quintet Rooting’ (QR), for rooting species trees that can be used with multi-locus datasets and take into consideration gene tree discordance due to ILS, as modeled by the multi-species coalescent (MSC) (Hudson, 1983) model. QR is based on the theoretical work by Allman, Degnan and Rhodes (henceforth, ‘ADR’) in Allman *et al.* (2011) that establishes that under the MSC, each five-leaf rooted species tree is identifiable from the distribution of the unrooted gene trees that it defines. ADR provide phylogenetic invariants and inequalities defined by the distribution on unrooted quintet trees that they prove hold under the MSC and that establish identifiability of any rooted five-leaf species tree. However, ADR do not suggest an estimation method that uses these invariants and inequalities. This study shows that QR is able to use these invariants and inequalities to more accurately root species trees under the MSC than previous methods.

## 2 Background

### 2.1 Previous methods for rooting trees

Rooting methods can generally be divided into three groups based on their assumptions about the input data. The technique most commonly used by biologists is outgroup rooting (Maddison *et al.*, 1984). In this approach, one or more species (called ‘outgroups’) that are distantly related to all the taxa in the original input set (called the ‘ingroup’) are added to this set, and a species tree is then estimated on the resulting expanded set. When the outgroup species are separated by an edge from the ingroup species, then the tree can be rooted on that edge (Kinene *et al.*, 2016). Although outgroup rooting is a natural and simple technique, it is not always reliable. If the outgroup species are too distant from the ingroup, they can function as ‘rogue taxa’ and estimation methods may be unable to find the correct placement for them in the final tree (and with sufficiently distant relationships, they can be placed on any edge in the tree with equal probability); this makes the root specification impossible. Conversely, when they are too closely related to the ingroup, this can produce very short edges in the species tree that also makes it difficult to find the correct root location (as accurate resolution of trees with very short edges is known to be difficult; Erdős *et al.*, 1999). Finally, sometimes a species that is supposed to be an outgroup is actually part of the ingroup. Moreover, previous work has shown that adding outgroups can even change the topology of the induced estimated tree on the ingroup species set, making the approach unreliable in some situations (Holland *et al.*, 2003). Therefore, choosing an appropriate outgroup needs prior biological knowledge as well as careful consideration of these scenarios, which may not be readily possible for some datasets and groups of species (Tarrío *et al.*, 2000; Wheeler, 1990).

Distance-based methods are another type of rooting method that can be used. These methods take an unrooted phylogeny with branch lengths as input and estimate the most likely location for the root based on specific assumptions about the model of evolution (Bettisworth and Stamatakis, 2021; Huelsenbeck *et al.*, 2002; Mai *et al.*, 2017; Tria *et al.*, 2017). Most methods of this type (e.g. midpoint rooting) are either based on molecular clock analysis or non-reversible models of DNA substitution. When a strict molecular clock holds, midpoint rooting and other distance-based methods can be statistically consistent and estimated root locations can be highly accurate. However, as the evolutionary rate deviates from strict molecular clock, midpoint rooting can perform poorly. Minimum ancestor deviation (MAD) (Tria *et al.*, 2017) and minimum variance rooting (MinVar) (Mai *et al.*, 2017) address this problem by minimizing a cost based on the deviation from the strict molecular clock. However, even MinVar and MAD can have poor accuracy as the deviation from the molecular clock and ILS level increases (Mai *et al.*, 2017).

Another method for rooting a species tree was introduced in Tian and Kubatko (2017). This method assumes that genes evolve within a species tree under the MSC and that sequences evolve within each gene tree under the Jukes and Cantor (1969) substitution model and with the strict molecular clock. This method takes a set of unrooted gene trees and their sequence alignments as input and uses a series of hypothesis tests on site pattern probabilities to infer a rooted quartet tree for every four species; it then uses these rooted quartet trees to root a given larger species tree. While the method performs well when the strict clock holds, its accuracy degrades as the clock is violated (Tian and Kubatko, 2017).

RootDigger (Bettisworth and Stamatakis, 2021) is an interesting new statistical method for rooting species trees. Given an unrooted species tree *T* and a sequence alignment, RootDigger computes the likelihood of each rooted version of *T* under a *non-reversible* model of evolution (specifically UNREST+T+I). In its default mode (i.e. ‘search’), RootDigger uses local search heuristics to find the most likely position for the root; for small enough trees, the ‘exhaustive’ mode can be used, which scores every rooted version to quantify root placement uncertainty by computing the likelihood weight ratio for placing the root on each branch of the tree. Because RootDigger is very recent, less is understood about its performance compared to the other methods we have discussed here.

Finally, STRIDE (Emms and Kelly, 2017) is relevant to rooting species trees when genes evolve with duplication and losses, so that the gene trees have multiple copies of the species. STRIDE uses properties about gene duplication and loss models to estimate the probability of the root being located on each edge in the species tree. However, by design, STRIDE cannot be used for rooting a species tree given single-copy gene trees.

### 2.2 The Allman, Degnan and Rhodes theory

Allman *et al.* (2011) (here given as ADR) provided one of the fundamental theorems underlying species tree estimation under the MSC: they proved that for any four species, the unrooted topology of the species tree is the same as the topology of the most probable unrooted gene tree. This theorem has been used to establish statistical consistency for quartet-based methods, such as ASTRAL, wQFM (Mahbub *et al.*, 2021) and the population tree in BUCKy (Larget *et al.*, 2010). Interestingly, Theorem 9 from ADR has received much less attention and (to the best of our knowledge) is not used in any species tree estimation method. This theorem states that every five-leaf *rooted* species tree topology is identifiable from the distribution of the *unrooted* gene tree topologies.

In deriving Theorem 9, ADR note that for any five species there are 105 possible rooted binary trees (i.e. there are 15 different unrooted binary gene tree topologies on 5 leaves, and each can be rooted on any of its 7 edges). Using the MSC, for any given rooted species tree on 5 leaves, ADR establish relationships (invariants and inequalities) between the probabilities for each of the 15 unrooted gene tree topologies, denoted by $u_{1},u_{2},\ldots ,u_{15}$. Every rooted five-leaf species tree has a particular topological shape (i.e. caterpillar, balanced or pseudo-caterpillar; Rosenberg, 2007), and ADR establish that the set of inequalities and invariants for a given rooted binary model species tree only depends on its shape and not on the numeric model parameters (i.e. branch lengths in coalescent units). Thus, the set of ADR invariants and inequalities fall into three categories, one for each shape. An example of these linear invariants and inequalities for each tree shape category is provided in Table 1. Theorem 9 in ADR establishes that under the MSC, these invariants and inequalities suffice to identify the rooted species tree and its internal branch lengths, for all rooted model species trees with five leaves. In other words, if the probability distribution on unrooted gene tree topologies is known exactly, then there will be exactly one rooted species tree topology that satisfies all the invariants and inequalities.

**Table 1.** Examples of invariants, inequalities and equivalence classes for rooted species trees of different categories according to ADR

	((((a,b),c),d),e)	(((a,b),c),(d,e))	(((a,b),(d,e)),c)
	Caterpillar	Balanced	Pseudo-caterpillar
Invariants	$\begin{array}{c} u_{14}-u_{15}=0\\ u_{11}-u_{15}=0\\ u_{10}-u_{15}=0\\ u_{8}-u_{15}=0\\ u_{7}-u_{15}=0\\ u_{6}-u_{9}=0\\ u_{5}-u_{12}=0\\ u_{4}-u_{13}=0\\ u_{2}-u_{3}+u_{9}-u_{12}=0 \end{array}$	$\begin{array}{c} u_{14}-u_{15}=0\\ u_{11}-u_{15}=0\\ u_{10}-u_{15}=0\\ u_{9}-u_{12}=0\\ u_{8}-u_{15}=0\\ u_{7}-u_{15}=0\\ u_{6}-u_{12}=0\\ u_{5}-u_{12}=0\\ u_{4}-u_{13}=0\\ u_{2}-u_{3}=0 \end{array}$	$\begin{array}{c} u_{14}-u_{15}=0\\ u_{12}-u_{15}=0\\ u_{10}-u_{15}=0\\ u_{9}-u_{15}=0\\ u_{8}-u_{11}=0\\ u_{7}-u_{15}=0\\ u_{6}-u_{15}=0\\ u_{5}-u_{15}=0\\ u_{4}-u_{13}=0\\ u_{2}-u_{3}=0 \end{array}$
Inequalities	$\begin{array}{c} u_{1}>u_{2},u_{4}>u_{5}>u_{7}\\ u_{3}>u_{2},u_{6}>u_{5}>u_{7} \end{array}$	$u_{1}>u_{2},u_{4}>u_{5}>u_{7}$	$u_{1}>u_{2},u_{4},u_{8}>u_{5}$
Equivalence Classes	$\left\{u_{1}\},\;\{u_{3}\right\}$ > > $\left\{u_{4},u_{13}\},\{u_{2}\},\{u_{6},u_{9}\right\}$ > $\left\{u_{5},u_{12}\right\}$ > $\left\{u_{7},u_{8},u_{10},u_{11},u_{14},u_{15}\right\}$	$\left\{u_{1}\right\}$ > $\left\{u_{2},u_{3}\},\{u_{4},u_{13}\right\}$ > $\left\{u_{5},u_{6},u_{9},u_{12}\right\}$ > $\left\{u_{7},u_{8},u_{10},u_{11},u_{14},u_{15}\right\}$	$\left\{u_{1}\right\}$ > $\left\{u_{2},u_{3}\},\{u_{4},u_{13}\},\{u_{8},u_{11}\right\}$ > $\left\{u_{5},u_{6},u_{7},u_{9},u_{10},u_{12},u_{14},u_{15}\right\}$

## 3 Quintet Rooting

### 3.1 General algorithmic design for QR

We propose a general class of methods for rooting that can be used when given an unrooted five-leaf species tree and a set of *k* unrooted five-leaf gene trees: First, compute the empirical probability distribution of the unrooted gene tree topologies and then score each rooted version of the given species tree to determine how well its topology fits the distribution as predicted by the ADR theory. Computing the (empirical) distribution on gene tree topologies is straightforward: divide the frequency for each unrooted gene tree topology by *k*. Scoring a rooted species tree, however, presents several non-trivial challenges. The first challenge is computational: each rooted species tree defines a different set of invariants and inequalities, and these must be calculated separately. The more significant challenge is how to define the fit between the ADR invariants and inequalities and a given rooted species tree so that the rooted species tree with the best fit is likely to be the true species tree. As we will see in the next section, defining the fit appropriately required that we correct for a topological bias in a naive definition of the fit.

In what follows, we will define cost functions for measuring the fit between the ADR invariants of a rooted five-leaf species tree and a given distribution of five-leaf unrooted gene trees, $g_{1},g_{2},\ldots ,g_{k}$. Then, given a cost function, distribution of five-leaf unrooted gene trees and an unrooted five-leaf tree *T*, we will seek the rooting of *T* that minimizes the cost. QR follows this design:

- Estimate the unrooted gene tree probability distribution $\vec{\hat{u}}=(\hat{u_{1}},\hat{u_{2}},\ldots ,\hat{u_{15}})$ from $g_{1},\ldots ,g_{k}$.
- For a given cost function $\mathrm{Cost}(R,\vec{\hat{u}})$ (for an example, see Equation 2), search all rooted versions of *T* to find $\hat{R}$ such that
  (1) $$\hat{R}=\underset{R}{\text{argmin}}C\mathrm{ost}(R,\vec{\hat{u}})$$

and return $\hat{R}$ as the rooted species tree.

In Section 3.3, we present extensions of this approach to enable us to root trees with more than five leaves.

### 3.2 Cost function

The ADR invariants lead to equivalence classes for *u_i_* values, examples of which are shown in Table 1. All the *u_i_* values in one equivalence class must be the same, and the ADR inequalities suggest certain inequality relationships across these classes. We define *C_R_* as the set of equivalence classes for a five-species rooted species tree *R*. For example, according to Table 1, the set of equivalence classes for the caterpillar tree $R_{1}=((((a,b),c),d),e)$ is given by

$$•\;\;C_{R_{1}}=\{\{u_{1}\},\{u_{2}\},\{u_{3}\},\{u_{4},u_{13}\},\{u_{6},u_{9}\},\{u_{5},u_{12}\},\{u_{7},u_{8},u_{10},u_{11},u_{14},u_{15}\}\}.$$

For two classes $c,c\prime \in C_{R}$, we write c>c′ if the inequalities for that specific tree state that each *u_i_* value in class *c* must be larger than each *u_i_* value in class c′. For instance, for *R*_1_, the inequalities in Table 1 requires that $\left\{u_{1}\}>\{u_{4},u_{13}\right\}$.

Each of the 105 possible five-species rooted trees has a unique set of equivalence classes, meaning that no two *C_R_*’s are exactly the same when considering the inequalities that hold among classes (see Supplementary Section S2 for a complete list of equivalence classes for all trees).

For a given vector of estimated gene tree probabilities $\vec{\hat{u}}=(\hat{u_{1}},\hat{u_{2}},\ldots ,\hat{u_{15}})$ and a rooted tree *R*, we seek to define a *cost* that measures the degree to which $\vec{\hat{u}}$ violates the ADR invariants and inequalities defined for that rooted tree *R*. Therefore, we seek to find a rooted species tree $\hat{R}$ for which this cost is minimized, which we will interpret as indicating that $\hat{R}$ best explains the given unrooted gene tree distribution according to ADR theory. If we let $\mathrm{Cost}(R,\vec{\hat{u}})$ denote this cost function, then we want $\mathrm{Cost}(R,\vec{\hat{u}})=0$ to indicate that the given gene tree distribution satisfies the ADR invariants and inequalities for that rooted species tree perfectly, and we want $\mathrm{Cost}(R,\vec{\hat{u}})>0$ to indicate that the gene tree distribution violates some of these ADR invariants and inequalities.

In Experiment 1 (see Section 4), we explored different cost functions on our training datasets (based on a mammalian simulation); here, we present the one we selected.

(2) $$\begin{array}{cc} \mathrm{Cost}(R,\vec{\hat{u}})= & \underset{\text{Invariants Penalty}}{\underset{︸}{\sum_{c\in C_{R}}\frac{1}{\left|c\right|}\sum_{u_{a},u_{b}\in c}\left|{\hat{u}}_{a}-{\hat{u}}_{b}\right|}}+\\ & \underset{\text{Inequalities Penalty}}{\underset{︸}{\sum_{c>c\prime \in C_{R}}\frac{1}{\left|c\prime \right|}\sum_{u_{a}\in c,u_{b}\in c\prime }\max(0,{\hat{u}}_{b}-{\hat{u}}_{a})}} \end{array}$$

The ‘Invariants Penalty’ component of Equation 2 considers a penalty for each pair ${\hat{u}}_{a},{\hat{u}}_{b}$ that are in the same equivalence class for *R* but have different values in $\vec{\hat{u}}$. Since membership in the same equivalence class indicates that *u_a_* = *u_b_*, the penalty for any pair ${\hat{u}}_{a},{\hat{u}}_{b}$ with different values is the magnitude of their difference. Summing these penalty terms is a natural way of penalizing the variation of *u_i’_*s inside equivalence classes. The ‘Inequalities Penalty’ component of Equation 2 is defined similarly, this time considering the inequalities between classes. For two classes *c* and c′ where c>c′, we expect to have ${\hat{u}}_{a}>{\hat{u}}_{b}$ for all $u_{a}\in c,u_{b}\in c\prime$ for the true tree (provided we have a good enough estimation of the probability distribution on quintet tree topologies). Therefore, we consider a penalty term equal to ${\hat{u}}_{b}-{\hat{u}}_{a}$ when this relationship is reversed (which means ${\hat{u}}_{b}-{\hat{u}}_{a}$ is a positive penalty). Note that all the individual penalty terms will be 0 when scoring the true species tree with respect to the true gene tree distribution.

**Normalization: correcting for bias.** The number of invariant penalty terms (i.e. $\left|{\hat{u}}_{a}-{\hat{u}}_{b}\right|$) in the cost function provided in Equation 2 for a rooted tree *R* is equal to $\sum_{c\in C_{R}}\left(\begin{array}{c} \left|c\right|\\ 2 \end{array}\right)$. Therefore, the number of invariant penalty terms for caterpillar, balanced and pseudo-caterpillar trees are 18, 23 and 31, respectively, which can be computed from class sizes in Table 1. Similarly, the number of inequality penalty terms for a tree *R* is equal to $\sum_{c>c\prime \in C_{R}}|c||c\prime |$. This leads to 28 inequality penalty terms for caterpillar trees, 44 for balanced trees and 54 for pseudo-caterpillar trees. Therefore, the overall number of penalty terms varies significantly between different tree shapes (46, 67 and 85, respectively), which could lead the algorithm to generally assign smaller costs to one tree category, and return an output tree from that category with much higher probability. In other words, using a cost function without modification would produce a ‘category bias’.

To alleviate this problem, we have used a normalization factor $\frac{1}{\left|c\right|}$ for the sum of invariant terms in a class *c* and a factor $\frac{1}{\left|c\prime \right|}$ for the sum of inequality terms between classes *c* and c′, where c′ is the class with smaller values. After including these factors, the *weighted* number of inequalities and invariants becomes 19, 20 and 19 for caterpillar, balanced and pseudo-caterpillar trees respectively. Hence, this results in roughly the same number of invariant and inequality penalty terms for each tree category.

### 3.3 Extending to larger trees

We have explored two ways of extending Quintet Rooing to trees with more than five leaves. Both operate by examining each of the 2n−3 possible ways to root the input unrooted *n*-leaf tree *T* (i.e. on each of the 2n−3 edges) and then evaluates the cost of the resultant rooted tree *R* by adding up the costs for a selected subset $Q^{*}$ of the rooted quintet trees within *R*. We describe this as a two-step process: (i) preprocessing: compute the cost for each rooted quintet tree using Equation 2 and (ii) for each of the 2n−3 candidate rooted trees *R* corresponding to *T*, compute the score of *R* as below:

(3) $$\mathrm{Score}(R,T)=\sum_{q\in Q^{*}}Cost(q,\hat{\vec{u_{q}}}),$$

where $Q^{*}$ is the selected subset of rooted quintet trees and $\hat{\vec{u_{q}}}$ is the probability distribution of unrooted five-species gene trees corresponding to a rooted quintet *q*. Thus, the two methods differ only in how the subset $Q^{*}$ of rooted quintet trees is defined. The first way looks at all possible rooted quintet trees, and so has $\Theta (n^{5})$ quintets to examine, but the second way looks at a carefully selected subset of *O*(*n*) quintets (where the selection is based on the unrooted species tree being evaluated). Moreover, by calculating the costs of the rooted quintet trees in a preprocessing step, the first approach uses $O(kn^{5})$ time and the second approach uses only *O*(*kn*) time, where there are *k* gene trees and *n* species. Thus, while the first approach has the advantage that it relies on more information, it is much less computationally tractable than the second approach. For the specific linear encoding approach we use, see Supplementary Section S4.

## 4 Experimental study design

**Overview.** In Experiment 1, we explored different cost functions for use in QR on *training* simulated datasets; this produced our selected cost function, presented in Section 3.2, which we used in all subsequent experiments for QR. In Experiments 2 and 3, we compared QR to four other rooting methods (three based on distances and RootDigger, which is based on gene sequence alignments as well as distances). In Experiment 2, we evaluated rooting methods on simulated ‘testing’ datasets with up to 30 leaves. In Experiment 3, we explored rooting methods on five-leaf subsets of an avian biological dataset.

We used the avian and mammalian simulated datasets from Mirarab *et al.* (2014b) and the biological avian dataset from Jarvis *et al.* (2014). We created subsets of 5 to 30 species each from these datasets to explore accuracy for rooting methods, using both true and estimated species trees. The model trees for the simulated datasets have model parameters (topology and coalescent unit branch lengths) based on the trees constructed on associated biological datasets, as described in Mirarab *et al.* (2014b) and below. We used both true species trees and estimated species trees, as computed using ASTRAL (Zhang *et al.*, 2018), and we reported the ‘clade distance’ between the estimated rooted trees and the true rooted trees as the error.

All experiments were run on the University of Illinois campus cluster, which limits each run to 4 h and explored the following questions: (i) How do different cost functions impact the accuracy of QR?, (ii) How does the accuracy of the species tree impact the relative and absolute accuracy of the different rooting methods? and (iii) How do the number of species and gene tree estimation error impact the relative and absolute accuracy of the different rooting methods?

**Evaluation**  **criteria.** We use the normalized clade distance between the estimated rooted tree *T* and the true rooted tree $T^{*}$ as the main measure of error, where clade distance is the natural extension of the standard Robinson and Foulds (1981) error rate that is used to evaluate methods for estimating unrooted trees. Thus, the normalized clade distance between two binary rooted trees $T^{*}$ and *T*, both on the same set of *n* leaves, is given by:

(4) $$\frac{\left|\mathrm{Clades}(T^{*})\backslash \mathrm{Clades}(T)|+|\mathrm{Clades}(T)\backslash \mathrm{Clades}(T^{*})\right|}{2n-4,}$$

where *Clades*(*t*) denotes the set of clades of the rooted tree *t*. Thus, the normalized clade distance is a value between 0 and 1 and indicates the fraction of the non-trivial clades in the estimated rooted tree that are not in the true rooted tree. When evaluating error in rooting the unrooted true tree *t*, another technique that can be used is the *root distance*, which is the distance in *t* between the edge containing the correct root location and the estimated root location. Letting *T* denote the result of applying a method to root *t* and letting $T^{*}$ denote the true rooted tree, the clade distance between *T* and $T^{*}$ is twice the root distance between *T* and $T^{*}$ (Lemma 1 in Supplementary Section S5). In our experiments, however, we are also interested in rooting estimated species trees, and for this case, we cannot use the root distance to measure error. For this reason, we use the clade distance (in its normalized form), which allows us to evaluate error in both cases. We also reported the proportion of test cases in which the tree was correctly rooted as a measure of accuracy for each method; results for this criterion show nearly identical relative performance as for the clade distance criterion, and are provided in Supplementary Section S6.2.

**Biological dataset**. We used the biological dataset studied in Jarvis *et al.* (2014) containing 48 avian species and 4 non-avian outgroups (American Alligator, Green Sea Turtle, Green Anole Lizard and Human). Jarvis *et al.* (2014) used 14 446 genes [8251 exons, 2516 introns and 3679 ultraconserved elements (UCEs)]; the main tree produced (the ‘TENT’, or total evidence nucleotide tree) was constructed using maximum likelihood and has branch lengths and branch support values. Because of the substantial levels of gene tree heterogeneity (e.g. every estimated gene tree was different from the estimated species tree) and because the estimated species tree had many very short branches suggestive of a rapid radiation which would produce high ILS, the avian dataset is considered to be a good example of a dataset with a high level of ILS (Jarvis *et al.*, 2014). The gene trees in this dataset exhibited exceptionally low branch support (on average about 32%), due to low rates of evolution in the exons and UCEs (Jarvis *et al.*, 2014), so that this is a challenging dataset for methods that are based on estimated gene trees.

We produced 12 subsets, each a random selection of 5 avian species. For each set of five avian species, we included any gene from the 14 446 genes that had all five species; this resulted in varying numbers of genes (ranging from approximately 10K to 13K genes) for each of the five-species subsets. For each selected subset of five species, we gave the unrooted TENT (restricted to those five species) to each of the rooting methods. We provided the published estimated gene trees to QR (after restriction to the selected five species), and we derived branch lengths on the five-leaf trees using the implied branch lengths in the TENT for the distance-based methods. To evaluate accuracy, we used the 48-species TENT, rooted at the edge leading to the outgroup, as the ‘true tree’.

**Simulated datasets**. We used mammalian simulated datasets for Experiment 1 (designing QR) and avian simulated datasets for Experiments 2 and 3 (evaluating QR in comparison to other methods). These datasets were generated by Mirarab *et al.* (2014b), and the true species trees, estimated and true gene trees and sequence alignments per gene are available at Mirarab *et al.* (2022).

Here, we briefly describe how Mirarab *et al.* (2014b) produced these datasets. The mammalian simulated datasets were evolved down a 37-species model tree based on a species tree constructed in Song *et al.* (2012) and the avian simulated datasets were evolved down a 48-species model tree based on the TENT constructed for Jarvis *et al.* (2014). [The Mirarab *et al.* (2014b) paper varied the ILS level by rescaling the branch lengths before simulating gene evolution, but here we only use the initial (default) species tree branch lengths.] Thousand gene trees were evolved down these model trees under the MSC, and the branch lengths were modified to create deviations from the molecular clock. Sequences of varying lengths were then evolved down each gene tree under a GTRGAMMA model of sequence evolution. Estimated gene trees were produced for each gene sequence alignment using maximum likelihood. Thus, the public repository contains true and estimated gene trees, true alignments and the true species trees (with branch lengths) for the mammalian and avian simulated datasets.

In Experiment 1, we only used true gene trees from the mammalian simulation, but in Experiment 2, we used both estimated and true gene trees from the avian simulation. We report the average gene tree estimation error (GTEE) for each model condition, where GTEE is the Robinson–Foulds error rates (i.e. the percentage of the non-trivial bipartitions in the true gene tree that are not found in the estimated gene tree). For the avian simulation, sequence lengths ranging from as long as 1600 down to 250 were provided, so that GTEE rates for the ML trees ranged from 30% to 67%. The ILS levels for these model conditions is reported using ‘average discordance’, or AD, defined as follows: AD is the average normalized bipartition distance between the true species tree and true gene trees. Thus, AD measures the percentage of bipartitions defined by internal branches in the species tree that are not in the true gene trees. The ILS level for the mammalian simulation used in our experiments is 29%, which is a moderate level of ILS, and the AD level for the avian simulation is 47%, which is moderately high.

To generate datasets with k-leaf trees, we randomly sampled k species from the species set and extracted the induced subtrees on these taxa from the model species tree and all gene trees in each model condition. For the five-species datasets, we generated 20 replicates (corresponding to the 20 replicates in the original datasets) of a dataset with 1000 random samples, and for all other datasets, we generated 20 replicates with 200 samples.

**Methods**. We compared QR to other rooting methods: midpoint rooting (Midpoint) as provided by the FastRoot (Mai *et al.*, 2017) package, minimum variance (MinVar) rooting (Mai *et al.*, 2017), MAD (Tria *et al.*, 2017) and RootDigger (Bettisworth and Stamatakis, 2021). The software versions and commands are provided in Supplementary Section S1. We did not include outgroup rooting, as it needs additional information about the taxa in the species set. We did not include STRIDE (Emms and Kelly, 2017), as it cannot be used on single-copy gene trees, and we did not include the rooting method from Tian and Kubatko (2017), as the software is not publicly available.

These methods require different types of input. The input to QR is a set of unrooted gene trees in addition to an unrooted species tree topology. The input to MinVar, MAD and Midpoint is an unrooted tree with branch lengths. Following the recommendations in Binet *et al.* (2016), who found that maximum likelihood was one of the two most accurate techniques for estimating branch lengths in species trees, we used RAxML (under GTRGAMMA) to estimate branch lengths on the given species trees, using a concatenated alignment of all gene sequences. Finally, the input to RootDigger is an unrooted tree with branch lengths as well as a multiple sequence alignment. To produce a multiple sequence alignment for RootDigger, we concatenated gene sequence alignments for all genes in a replicate. RootDigger has two modes of running that are called ‘search’ and ‘exhaustive’ modes. The default search mode provides a prediction of the root location quickly using heuristics, with early stopping on by default, and the exhaustive mode can be used to do a more thorough search and compute the confidence probabilities for the predicted root position. We ran RootDigger in both of these modes in our experiments, although the original paper only compared RootDigger in the ‘search’ mode with other methods. Our experiments included both the true unrooted species tree topology and an estimated unrooted species tree, computed by ASTRAL on the given gene trees.

## 5 Results and discussion

### 5.1 Experiment 1: Designing the cost function

Recall that QR can be used with any given cost function; hence, here, we compare four different cost functions to understand the impact of the cost function on the final accuracy. The final cost function, *Cost*_4_, is identical to the cost function given in Equation 2, and the first three cost functions are obtained by modifying *Cost*_4_. *Cost*_1_ only considers penalties for the invariants and not the inequalities, *Cost*_2_ considers both but does not normalize them, and *Cost*_3_ is similar to *Cost*_4_ in structure (i.e. it considers penalties for both invariants and inequalities) but uses a different normalization scheme. The equations for the first three cost functions are provided in Supplementary Section S3, but results comparing these cost functions are presented here.

**Fig. 1.**
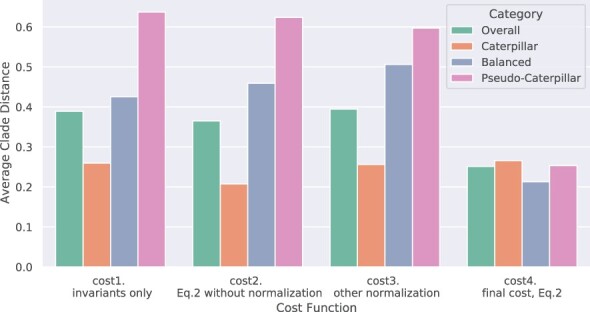
Average normalized clade distance for quintet rooting on mammalian simulated datasets using four different cost functions, across the different shape categories. The results are shown across 1000 sample five-leaf trees with 800 true gene trees. The ratio of caterpillar, balanced and pseudo-caterpillar trees in this dataset is 53.8%, 21.2% and 25%, respectively. The ILS level is 29% AD. This figure shows the effect of *Category Bias*, and the importance of how the cost function is defined. Based on this experiment, we selected *Cost*_4_ as our cost function

Figure 1 shows that QR using the first three cost functions produces biased results and overall lower accuracy: the rooting error rates (average normalized clade distance) on caterpillar trees are much lower than on balanced and pseudo-caterpillar trees. However, QR with *Cost*_4_ has approximately the same rooting error across the four different shape categories and overall has the lowest rooting error.

This bias results from the number of penalty terms (based on invariants and inequalities) for the caterpillar category being smaller than the number of penalty terms for the other categories, so that appropriate normalization is needed to eliminate the bias. Clearly not using the inequalities (*Cost*_1_) or not normalizing at all (*Cost*_2_) does not produce overall good results. A comparison between *Cost*_3_ and *Cost*_4_ is also interesting, as each uses both types of penalty terms and differ only in how they weight the inequalities. The approach used in *Cost*_4_ comes close to an equal weighted cost per category (see discussion in Section 3.2), explaining why using *Cost*_4_ results in lower overall error and greatly reduced bias.

### 5.2 Experiment 2: Evaluation on simulated datasets

Here, we compare QR to other rooting methods on 5-leaf and 10-leaf subtrees of the avian simulated datasets, with varying gene sequence lengths. We explore all conditions with both the true species tree and with the estimated species tree computed using ASTRAL. The distance-based methods and RootDigger were given branch lengths estimated by RAxML on the concatenated sequence alignments of all genes. Results shown for ‘true gene trees’ reflect performance when QR is given 1000 true gene trees and the other methods (which all require branch lengths on the species trees) are given branch lengths based on gene sequences of length 1600; all other conditions reflect performance given shorter sequences.

**Fig. 2.**
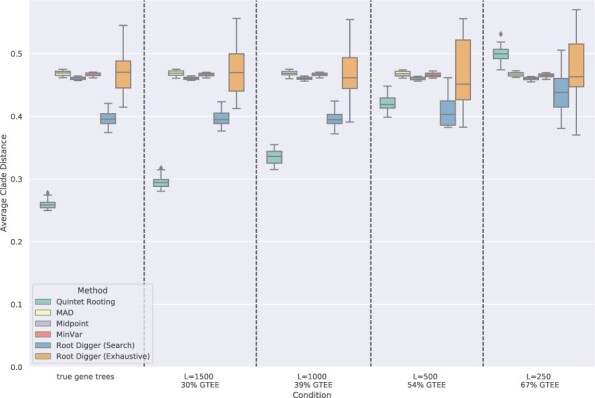
Average normalized clade distance of rooting methods on five-leaf subsets of the avian simulated datasets when given the true species tree topology. The number of genes is 1000 and the error bars are shown across 20 replicates, each on 1000 samples. Branch lengths are estimated using RAxML on the concatenated alignment (CA) of the gene sequence alignments. Results shown for ‘true gene trees’ reflect quintet rooting given 1000 true gene trees and the other methods given branch lengths estimated on the true species tree using gene sequences of length 1600

**Results for rooting**  **five-leaf trees.**  Figure 2 explores rooting error (computed using normalized clade distances) for three distance-based methods, two ways of running RootDigger and QR, when rooting five-leaf subtrees of the true species tree and varying the sequence length.

As expected, gene trees computed on shorter sequences have higher GTEE. Given true gene trees, QR has very low rooting error, but as GTEE increases its rooting error also increases. Even so, QR has much lower error than the other methods when GTEE is not high (i.e. GTEE <54%). However, for GTEE = 54%, QR is better than the distance-based methods but slightly worse than RootDigger run in its default search mode, and when GTEE = 67% then QR is less accurate than the other methods.

Other trends in Figure 2 are also worth noting. For example, the three distance-based methods (MAD, Midpoint and MinVar) do not seem impacted by the gene sequence length, so that at all sequence lengths the rooting error is very high. RootDigger run in search mode is impacted by sequence length, but not when run in exhaustive mode, and RootDigger run in exhaustive mode is much less accurate than RootDigger run in search mode for all sequence lengths (with big differences for longer sequences). Finally, although the differences between the distance-based methods are very small and to some extent depends on the model condition, Midpoint rooting is slightly more accurate than both MinVar and MAD, and when there was a difference between MinVar and MAD it tended to favor MinVar.

**Fig. 3.**
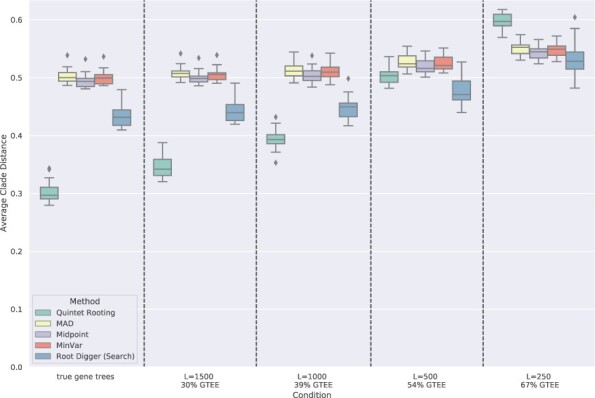
Average normalized clade distance on five-leaf avian simulated datasets by each rooting method given an estimated species tree computed by ASTRAL. The branch lengths on the estimated species tree are estimated using RAxML with the concatenated gene multiple sequence alignments. The results are averaged over 1000 sample five-species trees. The number of genes is 1000 and the error bars are shown across 20 replicates

Figure 3 shows a comparison between methods when rooting five-leaf estimated species trees computed by ASTRAL. In this experiment, we omitted RootDigger in exhaustive mode due to its poor accuracy when given the true species tree. Here, we observe the same trends as the experiments on the true species tree, with the relative performance between methods remaining the same. In particular, we still see QR having lower error than the other methods except for high GTEE (54%) and very high GTEE (67%), and Midpoint rooting more accurate than the other remaining methods. We also see that increases in sequence length improve accuracy for QR but do not impact the distance-based methods, and have only a minor impact on RootDigger.

**Fig. 4.**
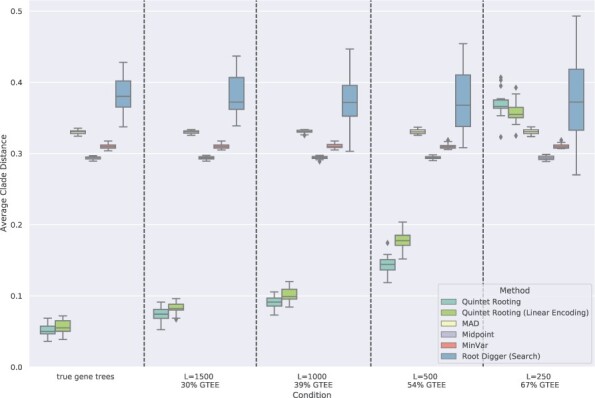
Average normalized clade distance on 10-leaf avian simulated datasets given the true (model) species tree by each rooting method. The results are averaged over 200 sample 10-species trees. The number of genes is 1000 and the error bars are shown across 20 replicates. The branch lengths are estimated using RAxML on the concatenated gene sequence alignments

**Results on rooting larger trees.** We now discuss results on rooting trees with 10–30 species. We begin with 10-leaf subtrees, comparing two ways of running QR, the three distance-based methods, and RootDigger in search mode. When rooting the true species tree (Fig. 4), both ways of running QR (i.e. looking at all five-leaf subtrees or the linear encoding) are more accurate than the other methods (except for the very high GTEE condition), and the linear encoding version is less accurate than the version that uses all five-leaf subtrees. Because both ways of running QR are (relatively) close in accuracy and have the same relative performance to other methods, we will refer to them jointly as ‘QR’ henceforth.

RootDigger is the least accurate, and the three distance-based methods are in between. For the highest GTEE rate (67%), QR is less accurate than the distance-based methods but still more accurate than RootDigger. The relative performance between distance-based methods is also noteworthy: Midpoint is the most accurate, followed by MinVar, and then by MAD. Changes in sequence length impact QR but not the distance-based methods, and impact RootDigger only slightly. Overall these trends are similar to trends observed on five-leaf trees, except that there are larger differences between the three distance-based methods and QR maintains its superior accuracy until the highest GTEE.

**Fig. 5.**
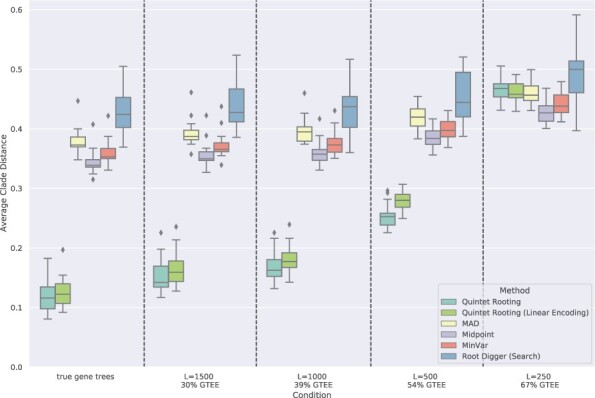
Average normalized clade distance on 10-leaf avian simulated datasets by each rooting method given an estimated species tree computed by ASTRAL. The branch lengths on the ASTRAL tree are estimated using RAxML with the concatenated gene multiple sequence alignments. The results are averaged over 200 sample 10-species trees. The number of genes is 1000 and the error bars are shown across 20 replicates

Figure 5 presents results when rooting 10-leaf trees computed by ASTRAL, instead of the true species trees. We see the same relative performance as before, but error rates are slightly higher than when rooting true species trees (which is unsurprising). And as when rooting true species trees, both ways of running QR are more accurate than the other methods except for the highest GTEE.

**Fig. 6.**
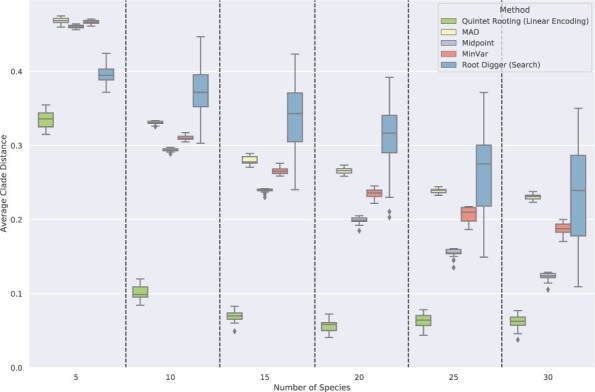
Average normalized clade distance on subsets of the avian simulated datasets with 5–30 leaves for each rooting method, given the true species tree topology and estimated gene trees with L = 1000 (39% GTEE). The branch lengths on the model tree are estimated using RAxML with the concatenated gene multiple sequence alignments. The results are averaged over 200 samples for each number of species. The number of genes is 1000 and the error bars are shown across 20 replicates

Results on trees with up to 30 leaves are shown in Figure 6, where we evaluate QR with the linear encoding in comparison to the other rooting methods when rooting subsets of the true species tree and given 1000 genes of length 1000. Note that QR with the linear encoding differs from default QR only on trees with more than five leaves, so that results for rooting five-leaf trees are identical to results in Figure 2. Here, we focus on the trends on the larger trees, and especially on results with more than 10 leaves.

QR using the linear encoding produces more accurate rootings than the other methods. Midpoint is the most accurate of the remaining methods, and RootDigger is the least accurate method (except when rooting five-leaf trees). All methods have relatively high error for five-leaf trees, but improve in accuracy as the tree size increases. Although there is a large gap between QR and the next best method starting at 10-leaf trees, the gap between QR and the other methods decreases with the tree size. However, even at 30 leaves, the error rate for QR is very low (6.2%) and about half that of the next best method, Midpoint (12.3%).

**Discussion of results on simulated datasets.** A comparison of results across these different conditions is informative. We see that QR run using all quintets is slightly more accurate than QR using the linear encoding and that both ways of running QR are much more accurate than all the other methods whenever GTEE is at most moderate. When GTEE is sufficiently high (i.e. at least 54%), then the relative performance between QR and the other methods depends on the number of species and whether true or estimated species trees are used. Specifically, QR maintains an advantage even at GTEE = 54% when rooting estimated species trees or when given 10-leaf trees. The distance-based methods were generally close in accuracy, but comparisons in terms of clade distances showed a consistent pattern of Midpoint somewhat more accurate than MinVar, and MinVar more accurate than MAD. Another trend that is consistently seen across all these conditions is that sequence length per gene always impacts QR, but does not impact distance-based methods, and has a very minor impact on RootDigger.

Some aspects of the relative performance, however, depended on the model condition. As an example, while RootDigger run in default mode was more accurate than the distance-based methods on 5-leaf trees, it was less accurate when rooting 10-leaf trees. We also saw that QR, using the linear encoding, was able to root trees with up to 30 leaves, and maintained an advantage over the other methods across all tree sizes we tested. However, the gap between QR and the next best method, Midpoint, decreased with the tree size. Finally, we observed that differences between methods were larger on true species trees than on estimated species trees, but relative performance remained the same.

In interpreting these trends, we make the following hypotheses. Clearly, QR is impacted by GTEE, which is why sequence length has an impact on it. However, the lack of impact on distance-based methods as well as the very minor impact on RootDigger (which also uses branch lengths) suggest that using 1000 genes is sufficient, even for short gene alignments, to produce a reasonable estimate of the branch lengths of the given species tree. The improvement in accuracy for the distance-based methods and even RootDigger as the tree size increases suggests that these methods benefit from denser taxon sampling, which may break up long branches and possibly improves branch length estimation.

### 5.3 Experiment 3: Evaluation on the biological dataset

Because we observed that QR is impacted by gene tree estimation error, we selected a biological dataset in which this was likely to be a challenge for it: the Avian Phylogenomics project dataset studied in Jarvis *et al.* (2014). Gene tree branch support (measured using bootstrapping) was on average about 32% (Mirarab *et al.*, 2014b), resulting from low phylogenetic signal in the gene sequences, and suggesting that the gene trees likely had high gene tree estimation error (Mirarab *et al.*, 2014b). Evaluating how well QR performed on this dataset would give us an estimate of its reliability under very challenging conditions. [Here, we note that many other phylogenomic datasets are not as challenging; for example, the average branch support in the Thousand Plant Transcriptome project from Wickett *et al.* (2014) was much higher.]

**Fig. 7.**
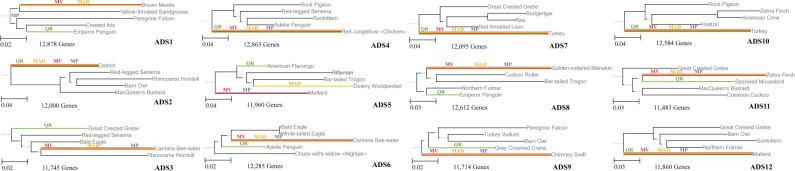
Analyses on the five-leaf subsets of the total evidence nucleotide tree (TENT) computed on the avian biological dataset by Jarvis *et al.* (2014). The branch lengths are obtained from the published TENT species tree using Dendropy (Sukumaran and Holder, 2010). The branch selected as the root by each method is color-coded. The number of genes used in each analysis (defined to be those genes that have all five of the selected species) is also shown beside each figure. Results are shown for quintet rooting (QR), midpoint (MP), MinVar (MV) and minimum ancestor deviation (MAD). The trees are visualized using ETEToolkit v3 (Huerta-Cepas *et al.*, 2016)

We selected 12 random subsets of 5 avian species each from the maximum likelihood ‘TENT’ tree computed on the genome-scale data and used the outgroups to root the trees as the ‘reference rooted tree’ for evaluation purposes. The normalized clade distances for QR and the three distance-based rooting methods on the 12 avian subtrees are provided in Table 2, with the trees shown in Figure 7. The two methods with the lowest average error are QR and Midpoint, but MinVar is close behind and MAD is in last place. One of the striking observations is that the distance-based methods tend to root the tree on the longest branch in the tree, but this is not true for QR. We also see that there are datasets for which no method is able to find the correct root (see ADS8 and ADS11, for example).

**Table 2.** Normalized clade distances are shown for Quintet Rooting, Midpoint, MinVar and minimum ancestor deviation (MAD) for rooting five-leaf subtrees of the TENT avian tree

Dataset	No. of genes	Quintet rooting	Midpoint	MinVar	MAD
ADS1	12 878	0.67	0.00	0.33	0.33
ADS2	12 000	0.00	0.00	0.00	0.00
ADS3	11 745	0.00	1.00	1.00	1.00
ADS4	12 863	0.00	0.00	0.00	0.00
ADS5	11 960	0.33	0.00	0.00	1.00
ADS6	12 285	0.33	0.67	0.67	0.67
ADS7	12 095	0.00	0.00	0.00	0.00
ADS8	12 612	0.33	0.33	0.33	0.33
ADS9	11 714	0.33	0.00	0.00	0.00
ADS10	12 584	0.00	0.00	0.00	0.00
ADS11	11 483	0.67	0.67	0.67	0.67
ADS12	11 860	0.00	0.00	0.00	0.00
Average	∼12 173	0.22	0.22	0.25	0.33

*Note*: The reference tree is the TENT tree from Jarvis *et al.* (2014), rooted on the edge leading to the outgroups.

The trends on these biological datasets are consistent with results on the simulated datasets. As noted, the biological dataset gene trees have low bootstrap support indicating that for these datasets the gene tree estimation error is probably in the 50–80% range. Thus, the corresponding cases among the simulated data are when GTEE is high or very high. On the biological dataset, QR was in first place but tied with midpoint rooting (MP), with MinVar somewhat less accurate than QR and MP, and then MAD in last place. On simulated datasets, we saw QR in first place except when gene tree estimation error is high or very high. The relative performance between the three distance-based methods is also similar on the biological as well as simulated data, since MP was first in the simulated data, followed by MinVar and then by MAD, which is what we see here.

## 6 Conclusions

We have presented QR, a polynomial time method for rooting a given species tree that uses phylogenetic invariants and inequalities established by Allman *et al.* (2011) under the MSC model. The trends observed on both biological and simulated data suggest that QR is a promising approach to rooting species trees under a range of model conditions, generally more accurate than all the tested competing methods except when GTEE is very high, in which case all methods have poor accuracy.

The study suggests several directions for future work. For example, we have not established whether QR is a statistically consistent method for rooting species trees, not even for the simplest case of five-leaf trees. We conjecture that this is the case, but a proof is needed. In addition, alternative cost functions need to be explored to determine if even more accurate methods can be developed and established statistically consistent.

Further study under a wider range of conditions is also needed. In particular, although QR (using the linear encoding) was able to maintain an accuracy advantage over the competing methods with up to 30 leaves, the gap between QR and the next most accurate method had reduced, and it is possible that on much larger trees QR might be less accurate than other methods. Thus, evaluating QR and other rooting methods on larger trees is needed. Evaluating methods when given larger numbers of genes is another important direction to consider, especially since many phylogenomic studies use 1000 or more genes to estimate species trees (e.g. Jarvis *et al.*, 2014 used 14 000 genes). Future work should also evaluate conditions with varying levels of ILS, and the model conditions we explored were based on datasets with moderately high ILS. Deviation from the strict molecular clock has the potential to impact all methods, even if the main impact would be on methods that try to minimize the deviation from the clock or from a relaxed clock model, and its impact should be explored systematically. Another question is why RootDigger performed poorly in this study. Since RootDigger depends on likelihood calculations, one possibility is inadequate search of the parameter space, but another possibility is model misspecfication. All the methods we explore depend on stochastic models of evolution, either for computing distances or for estimating gene trees; hence, the impact of model misspecification should be explored more generally.

The theoretical foundations for the method, provided in Allman *et al.* (2011), are specific to the MSC model, but this does not mean that the approach would not perform well under other conditions, such as cases where there is (for example) HGT or GDL. Future work should evaluate QR under a wider range of model conditions with these and other causes for gene tree discord.

Finally, a closely related problem is the estimation of the rooted species tree from a set of unrooted gene trees, under the MSC. This is a strictly harder problem than estimating a rooted species tree from *rooted* gene trees under the MSC, which has been addressed by methods such as MP-EST (Liu *et al.*, 2010). Hence, this is another direction for future work.

*Financial Support*: none declared.

*Conflict of Interest*: none declared.

## Supplementary Material

btac224_Supplementary_Data
