# Supplementary material for: Quintet Rooting: rooting species trees under the multi-species coalescent model
Source: Bioinformatics. 2022 Jun 27;38(Suppl 1):i109–17. doi: 10.1093/bioinformatics/btac224 (PMC9236578; doi:10.1093/bioinformatics/btac224)
Supplement: btac224_Supplementary_Data [file btac224_supplementary_data.pdf]

Supplementary Material for "Quintet Rooting: Rooting Species Trees under the Multi-Species Coalescent Model"

Yasamin Tabatabaee, Kowshika Sarker, Tandy Warnow

Department of Computer Science, University of Illinois at Urbana-Champaign

Contents

S1 Details about the Experimental Study 3
- S1.1 Methods for rooting trees . . . 3
- S1.2 Other commands . . . 3
- S1.3 Biological dataset analyses . . . 4

S2 List of Equivalence Classes of Rooted 5-taxon Trees 6
- S2.1 Caterpillar Trees . . . 6
- S2.2 Pseudo-caterpillar Trees . . . 10
- S2.3 Balanced Trees . . . 11

S3 Cost functions 12

S4 Linear Encoding of Trees by Quintets 13

S5 Relationship between Root Distance and Clade Distance 14

S6 Additional Results 15
- S6.1 Rooting larger trees . . . 15
- S6.2 Rooting Accuracy . . . 16

List of Figures

S1 Linear encoding of trees by quintets 13
S2 Example relating clade distance to root distance 14
S3 Error rates for rooting subtrees of the simulated avian model species tree with 5 to 30 leaves. 15
S4 Correct rooting proportion given true 5-leaf species trees 16
S5 Correct rooting proportion given estimated 5-leaf species trees 17
S6 Correct rooting proportion given true 10-leaf species trees 18
S7 Correct rooting proportion given estimated 10-leaf species trees 19

List of Tables

S1 Avian biological tree subsets in newick format 5
S2 Equivalence classes for caterpillar trees 6
S3 Equivalence classes for pseudo-caterpillar trees 10
S4 Equivalence classes for balanced trees 11

S1 Details about the Experimental Study

S1.1 Methods for rooting trees

Minimum Ancestor Deviation. The python script for MAD (v2.2) software is available at https://www.mikrobio.uni-kiel.de/de/ag-dagan/ressourcen. The command we used is:

```
python3 mad.py <input-tree.tre> -n
```

Minimum Variance Rooting. MinVar and Midpoint rooting are both available as part of the FastRoot (v1.5) python package at https://github.com/uym2/MinVar-Rooting. The command we used is:

```
python3 FastRoot.py -m MV -i <input-tree.tre> -o <output-tree.tre>
```

Midpoint Rooting. The command we used is:

```
python3 FastRoot.py -m MP -i <input-tree.tre> -o <output-tree.tre>
```

Quintet Rooting. Quintet Rooting (v1.0) is available at https://github.com/ytabatabaee/Quintet-Rooting. The option `-sm LE` can be used to sample a sparse set of quintets using a linear encoding and runs considerably faster than the default mode. We used the following command:

```
python3 quintet_rooting.py -t <species-topology.tre>
        -g <input-genes.tre>  -o <output.tre> [-sm LE]
```

RootDigger. We used RootDigger (v1.7.0), which is available at https://github.com/computations/root_digger. This method has two modes, the default is search and the other can be identified with `--exhaustive`. We used the following command:

```
./rd --msa <msa-file.fasta> --tree <input-tree.tre>
     --seed 4321 [--exhaustive]
```

S1.2 Other commands

Species tree estimation. We used ASTRAL (v5.7.8) to estimate species trees. ASTRAL is available at https://github.com/smirarab/ASTRAL as a jar file. The command we used is:

```
java -jar astral.5.7.8.jar -i <input-genes.tre> -o <output.tre>
```

Branch length estimation. We used RAxML (v8.2.12) to estimate branch lengths on given species trees, using the concatenated alignment. RAxML is available at https://github.com/stamatak/standard-RAxML. We used the following command:

```
raxmlHPC-PTHREADS -f e -t species.tre -m GTRGAMMA -s alignment.
    fasta -n RES -p 4321 -T 16
```

Calculating clade distance The following function was used to compute the normalized clade distance between two *rooted* trees, where `t1` and `t2` are Dendropy rooted `Tree` objects with `n` leaves, constrained to the same set of taxa (i.e. sharing the same `TaxonNamespace` object).

```
def normalized_clade_distance(t1, t2, n):
    t1.encode_bipartitions()
    t2.encode_bipartitions()
    return dendropy.calculate.treecompare.symmetric_difference(t1,
   t2) / (2n - 4)
```

Random subset selection We selected random subsets of taxa in a tree using Python's default `random` module with the following command:

```
sample_taxa = random.sample(taxon_set, n)
```

where `taxon_set` is the list of all taxa in the original tree and `n` is the size of the subset tree.

Extracting induced trees After selecting subsets of taxa, we extracted the induced subset trees using Dendropy's `extract_tree_with_taxa_labels` on a `Tree` object using the following command:

```
subtree = tree.extract_tree_with_taxa_labels(labels=sample_taxa,
   suppress_unifurcations=True)
```

where `tree` is the original unrooted species tree or the corresponding unrooted gene trees and `sample_taxa` are the labels selected at previous step. After constraining the tree to the selected taxa, the `suppress_unifurcations` option removes nodes with degree two and connects their two adjacent nodes and adjusts the edge lengths accordingly [5].

S1.3 Biological dataset analyses

Experiment 3 is based on analyses of the avian phylogenomics project [4] dataset, where we attempted to root 5-leaf subsets of the TENT (total evidence nucleotide tree). The data repository for the avian phylogenomics project (available at [3]) contains: (a) alignments for each gene, (b) maximum likelihood gene trees, and (c) the TENT topology as well as branch lengths. We used these for our rooting analyses.

We selected 12 different 5-leaf subsets of the ingroup species from the TENT, using the random subset selection method described above and obtained the 5-leaf induced subtrees of the species tree and gene trees as explained above. We used the branch lengths values produced by `extract_tree_with_taxa_labels` function, that adjusts the branch lengths after suppressing unifurcations from the induced subtrees. [5].

We used a similar constraining and suppressing approach to get induced subtrees on the 14,446 gene trees (including all exons, introns and UCEs). Since some gene trees had missing taxa, for each data subset, only the gene trees that had all taxa in that dataset were considered, reducing the number of genes used in each analysis to a value ranging from 11k to 13k.

The trees in Figure 7 (in the main paper) are visualized using the online phylogenetic tree viewer tool from ETEToolkit v3 [2] and are provided below in newick format (in rooted form, as rooted by the outgroup species). The mapping between the 5-letter codes of the leaves in these trees and the actual species names are available on the dataset repository at http://gigadb.org/dataset/101041 in the `Scripts/namemap/name.csv` directory in the `Scripts.tar.gz` file.

Table S1: 12 avian biological 5-leaf tree subsets from the Avian Phylogenomics project dataset studied in [4] in newick format. The mapping between 5-letter codes for the leaves in the trees and actual species names is provided at the original dataset repository.

Dataset	Species Subtree (in newick format)
ADS1	((MESUN:0.08694290426167792,PTEGU:0.071415818897068)100:0.00514859723893367,(FALPE:0.08306071570403102,(NIPNI:0.0423883355661155,APTFO:0.03349709647931403)100:0.005206840108496662)93:0.0032472606038615546)100:0.06743839999387245;
ADS2	(STRCA:0.12274985497254273,((CARCR:0.05643578593379514,(BUCRH:0.10530648854898142,TYTAL:0.06741501268170091)94:0.0014309181653352882)100:0.006338016412161908,CHLUN:0.07664747894993916)100:0.06853118805605843)100;
ADS3	(PODCR:0.07220590793996046,(CARCR:0.05643578593379514,(HALLE:0.045566642130790286,(MERNU:0.10658290270301818,BUCRH:0.0994958728004391)100:0.0064298085261464575)100:0.0008117253877311482)100:0.0074308044743478935)100:0.06743839999387245;
ADS4	((COLLI:0.09853337060176218,(CARCR:0.06061932980428148,(EURHE:0.0976862721822968,PYGAD:0.04011775509160112)49:0.0011991271659342246)93:0.0032472606038615546)100:0.03161522291278337,GALGA:0.19209772733128544)100:0.035823177081089085;
ADS5	((PHORU:0.045008036433236975,(ACACH:0.12720876507668982,(APAVI:0.10459378362169088,PICPU:0.1493872121305442)80:0.004687827119484025)100:0.0074308044743478935)100:0.03161522291278337,ANAPL:0.1400222823017829)100:0.035823177081089085;
ADS6	((((HALLE:0.0009993079648479574,HALAL:0.0011481494716151777)100:0.04456733416594233,MERNU:0.11301271122916463)100:0.004995269258217487,PYGAD:0.041316882257535346)93:0.0021544725416755696,CAPCA:0.07442083096028874)100:0.06853118805605843;
ADS7	((PODCR:0.07220590793996046,((MELUN:0.05843745032225126,NESNO:0.04201569459493401)100:0.061839565146662875,GAVST:0.04769342999296146)93:0.0032472606038615546)100:0.03161522291278337,MELGA:0.19796647631793823)100:0.035823177081089085;
ADS8	((MANVI:0.12207721157673541,(LEPDI:0.06682837969931472,APAVI:0.10585054810092069)100:0.0034310626402542107)100:0.004183543870486339,(FULGL:0.03407937342341265,APTFO:0.03230433568829369)100:0.006399600899517001)93:0.07068566059773401;
ADS9	(((FALPE:0.07887717183354469,(CATAU:0.03200310265259699,TYTAL:0.06803420545930505)100:0.0008117253877311482)100:0.00530612570207519,BALRE:0.05940380102308789)88:0.0010318907100867183,CHAPE:0.11587887735386451)100:0.06853118805605843;
ADS10	((COLLI:0.09853337060176218,((TAEGU:0.0664816204499164,CORBR:0.03848548508393826)100:0.1021859557356423,OPHHO:0.07635447103047725)88:0.0021246787722727033)100:0.03161522291278337,MELGA:0.19796647631793823)100:0.035823177081089085;
ADS11	(PODCR:0.07220590793996046,((TAEGU:0.16336145048348355,COLST:0.12215804393381353)100:0.006338016412161908,(CHLUN:0.07443664039789928,CUCCA:0.1112755055198169)100:0.0022108385520398783)100:0.001092788062185985)100:0.06743839999387245;
ADS12	((PODCR:0.07220590793996046,(TYTAL:0.07302947471752254,(EURHE:0.0976862721822968,FULGL:0.03927984715699542)49:0.0011991271659342246)93:0.0032472606038615546)100:0.03161522291278337,ANAPL:0.1400222823017829)100:0.035823177081089085;

S2 List of Equivalence Classes of Rooted 5-taxon Trees

S2.1 Caterpillar Trees

Table S2: Equivalence classes for caterpillar 5-taxon rooted species trees. The u_is are named according to the unrooted trees in Table 5 in [1]. A script for generating these classes is available in the Quintet Rooting software repository in Github.

Tree	Equivalence Classes
R_1 $((((a,b),c),d),e)$	$\{u_1\} > \{u_4, u_{13}\}$<br>$\{u_2\} > \{u_5, u_{12}\} > \{u_7, u_8, u_{10}, u_{11}, u_{14}, u_{15}\}$<br>$\{u_3\} > \{u_6, u_9\}$
R_2 $((((a,b),c),e),d)$	$\{u_1\} > \{u_4, u_{13}\}$<br>$\{u_3\} > \{u_6, u_9\} > \{u_7, u_8, u_{10}, u_{11}, u_{14}, u_{15}\}$<br>$\{u_2\} > \{u_5, u_{12}\}$
R_3 $((((a,b),d),c),e)$	$\{u_2\} > \{u_7, u_{14}\}$<br>$\{u_1\} > \{u_8, u_{11}\} > \{u_4, u_5, u_{10}, u_{12}, u_{13}, u_{15}\}$<br>$\{u_3\} > \{u_9, u_6\}$
R_4 $((((a,b),d),e),c)$	$\{u_2\} > \{u_7, u_{14}\}$<br>$\{u_3\} > \{u_6, u_9\} > \{u_4, u_5, u_{10}, u_{12}, u_{13}, u_{15}\}$<br>$\{u_1\} > \{u_8, u_{11}\}$
R_5 $((((a,b),e),c),d)$	$\{u_3\} > \{u_{10}, u_{15}\}$<br>$\{u_1\} > \{u_8, u_{11}\} > \{u_4, u_6, u_7, u_9, u_{13}, u_{14}\}$<br>$\{u_2\} > \{u_5, u_{12}\}$
R_6 $((((a,b),e),d),c)$	$\{u_3\} > \{u_{10}, u_{15}\}$<br>$\{u_2\} > \{u_5, u_{12}\} > \{u_4, u_6, u_7, u_9, u_{13}, u_{14}\}$<br>$\{u_1\} > \{u_8, u_{11}\}$
R_7 $((((a,c),b),d),e)$	$\{u_4\} > \{u_1, u_{13}\}$<br>$\{u_5\} > \{u_2, u_{12}\} > \{u_7, u_8, u_{10}, u_{11}, u_{14}, u_{15}\}$<br>$\{u_6\} > \{u_3, u_9\}$
R_8 $((((a,c),b),e),d)$	$\{u_4\} > \{u_1, u_{13}\}$<br>$\{u_6\} > \{u_3, u_9\} > \{u_7, u_8, u_{10}, u_{11}, u_{14}, u_{15}\}$<br>$\{u_5\} > \{u_2, u_{12}\}$
R_9 $((((a,c),d),b),e)$	$\{u_5\} > \{u_8, u_{15}\}$<br>$\{u_4\} > \{u_7, u_{10}\} > \{u_1, u_2, u_{11}, u_{12}, u_{13}, u_{14}\}$<br>$\{u_6\} > \{u_3, u_9\}$
R_{10} $((((a,c),d),e),b)$	$\{u_5\} > \{u_8, u_{15}\}$<br>$\{u_6\} > \{u_3, u_9\} > \{u_1, u_2, u_{11}, u_{12}, u_{13}, u_{14}\}$<br>$\{u_4\} > \{u_7, u_{10}\}$
R_{11} $((((a,c),e),b),d)$	$\{u_6\} > \{u_{11}, u_{14}\}$<br>$\{u_4\} > \{u_7, u_{10}\} > \{u_1, u_3, u_8, u_9, u_{13}, u_{15}\}$<br>$\{u_5\} > \{u_2, u_{12}\}$
R_{12} $((((a,c),e),d),b)$	$\{u_6\} > \{u_{11}, u_{14}\}$<br>$\{u_5\} > \{u_2, u_{12}\} > \{u_1, u_3, u_8, u_9, u_{13}, u_{15}\}$<br>$\{u_4\} > \{u_7, u_{10}\}$
R_{13} $((((a,d),b),c),e)$	$\{u_7\} > \{u_2, u_{14}\}$<br>$\{u_8\} > \{u_1, u_{11}\} > \{u_4, u_5, u_{10}, u_{12}, u_{13}, u_{15}\}$<br>$\{u_9\} > \{u_3, u_6\}$
R_{14} $((((a,d),b),e),c)$	$\{u_7\} > \{u_2, u_{14}\}$<br>$\{u_9\} > \{u_3, u_6\} > \{u_4, u_5, u_{10}, u_{12}, u_{13}, u_{15}\}$<br>$\{u_8\} > \{u_1, u_{11}\}$

Tree	Equivalence Classes
R_{15} $((((a,d),c),b),e)$	$\{u_8\} > \{u_5,u_{15}\}$<br>$\{u_7\} > \{u_4,u_{10}\} > \{u_1,u_2,u_{11},u_{12},u_{13},u_{14}\}$<br>$\{u_9\} > \{u_3,u_6\}$
R_{16} $((((a,d),c),e),b)$	$\{u_8\} > \{u_5,u_{15}\}$<br>$\{u_9\} > \{u_3,u_6\} > \{u_1,u_2,u_{11},u_{12},u_{13},u_{14}\}$<br>$\{u_7\} > \{u_4,u_{10}\}$
R_{17} $((((a,d),e),b),c)$	$\{u_9\} > \{u_{12},u_{13}\}$<br>$\{u_7\} > \{u_4,u_{10}\} > \{u_2,u_3,u_5,u_6,u_{14},u_{15}\}$<br>$\{u_8\} > \{u_1,u_{11}\}$
R_{18} $((((a,d),e),c),b)$	$\{u_9\} > \{u_{12},u_{13}\}$<br>$\{u_8\} > \{u_1,u_{11}\} > \{u_2,u_3,u_5,u_6,u_{14},u_{15}\}$<br>$\{u_7\} > \{u_4,u_{10}\}$
R_{19} $((((a,e),b),c),d)$	$\{u_{10}\} > \{u_3,u_{15}\}$<br>$\{u_{11}\} > \{u_1,u_8\} > \{u_4,u_6,u_7,u_9,u_{13},u_{14}\}$<br>$\{u_{12}\} > \{u_2,u_5\}$
R_{20} $((((a,e),b),d),c)$	$\{u_{10}\} > \{u_3,u_{15}\}$<br>$\{u_{12}\} > \{u_2,u_5\} > \{u_4,u_6,u_7,u_9,u_{13},u_{14}\}$<br>$\{u_{11}\} > \{u_1,u_8\}$
R_{21} $((((a,e),c),b),d)$	$\{u_{11}\} > \{u_6,u_{14}\}$<br>$\{u_{10}\} > \{u_4,u_7\} > \{u_1,u_3,u_8,u_9,u_{13},u_{15}\}$<br>$\{u_{12}\} > \{u_2,u_5\}$
R_{22} $((((a,e),c),d),b)$	$\{u_{11}\} > \{u_6,u_{14}\}$<br>$\{u_{12}\} > \{u_2,u_5\} > \{u_1,u_3,u_8,u_9,u_{13},u_{15}\}$<br>$\{u_{10}\} > \{u_4,u_7\}$
R_{23} $((((a,e),d),b),c)$	$\{u_{12}\} > \{u_9,u_{13}\}$<br>$\{u_{10}\} > \{u_4,u_7\} > \{u_2,u_3,u_5,u_6,u_{14},u_{15}\}$<br>$\{u_{11}\} > \{u_1,u_8\}$
R_{24} $((((a,e),d),c),b)$	$\{u_{12}\} > \{u_9,u_{13}\}$<br>$\{u_{11}\} > \{u_1,u_8\} > \{u_2,u_3,u_5,u_6,u_{14},u_{15}\}$<br>$\{u_{10}\} > \{u_4,u_7\}$
R_{25} $((((b,c),a),d),e)$	$\{u_{13}\} > \{u_1,u_4\}$<br>$\{u_{12}\} > \{u_2,u_5\} > \{u_7,u_8,u_{10},u_{11},u_{14},u_{15}\}$<br>$\{u_9\} > \{u_3,u_6\}$
R_{26} $((((b,c),a),e),d)$	$\{u_{13}\} > \{u_1,u_4\}$<br>$\{u_9\} > \{u_3,u_6\} > \{u_7,u_8,u_{10},u_{11},u_{14},u_{15}\}$<br>$\{u_{12}\} > \{u_2,u_5\}$
R_{27} $((((b,c),d),a),e)$	$\{u_{12}\} > \{u_{10},u_{11}\}$<br>$\{u_{13}\} > \{u_{14},u_{15}\} > \{u_1,u_2,u_4,u_5,u_7,u_8\}$<br>$\{u_9\} > \{u_3,u_6\}$
R_{28} $((((b,c),d),e),a)$	$\{u_{12}\} > \{u_{10},u_{11}\}$<br>$\{u_9\} > \{u_3,u_6\} > \{u_1,u_2,u_4,u_5,u_7,u_8\}$<br>$\{u_{13}\} > \{u_{14},u_{15}\}$
R_{29} $((((b,c),e),a),d)$	$\{u_9\} > \{u_7,u_8\}$<br>$\{u_{13}\} > \{u_{14},u_{15}\} > \{u_1,u_3,u_4,u_6,u_{10},u_{11}\}$<br>$\{u_{12}\} > \{u_2,u_5\}$
R_{30} $((((b,c),e),d),a)$	$\{u_9\} > \{u_7,u_8\}$<br>$\{u_{12}\} > \{u_2,u_5\} > \{u_1,u_3,u_4,u_6,u_{10},u_{11}\}$<br>$\{u_{13}\} > \{u_7,u_8\}$

Tree	Equivalence Classes
R_{31} $((((b,d),a),c),e)$	$\{u_{14}\} > \{u_2,u_7\}$<br>$\{u_{11}\} > \{u_1,u_8\} > \{u_4,u_5,u_{10},u_{12},u_{13},u_{15}\}$<br>$\{u_6\} > \{u_3,u_9\}$
R_{32} $((((b,d),a),e),c)$	$\{u_{14}\} > \{u_2,u_7\}$<br>$\{u_6\} > \{u_3,u_9\} > \{u_4,u_5,u_{10},u_{12},u_{13},u_{15}\}$<br>$\{u_{11}\} > \{u_1,u_8\}$
R_{33} $((((b,d),c),a),e)$	$\{u_{11}\} > \{u_{10},u_{12}\}$<br>$\{u_{14}\} > \{u_{13},u_{15}\} > \{u_1,u_2,u_4,u_5,u_7,u_8\}$<br>$\{u_6\} > \{u_3,u_9\}$
R_{34} $((((b,d),c),e),a)$	$\{u_{11}\} > \{u_{10},u_{12}\}$<br>$\{u_6\} > \{u_3,u_9\} > \{u_1,u_2,u_4,u_5,u_7,u_8\}$<br>$\{u_{14}\} > \{u_{13},u_{15}\}$
R_{35} $((((b,d),e),a),c)$	$\{u_6\} > \{u_4,u_5\}$<br>$\{u_{14}\} > \{u_{13},u_{15}\} > \{u_2,u_3,u_7,u_9,u_{10},u_{12}\}$<br>$\{u_{11}\} > \{u_1,u_8\}$
R_{36} $((((b,d),e),c),a)$	$\{u_6\} > \{u_4,u_5\}$<br>$\{u_{11}\} > \{u_1,u_8\} > \{u_2,u_3,u_7,u_9,u_{10},u_{12}\}$<br>$\{u_{14}\} > \{u_{13},u_{15}\}$
R_{37} $((((b,e),a),c),d)$	$\{u_{15}\} > \{u_3,u_{10}\}$<br>$\{u_8\} > \{u_1,u_{11}\} > \{u_4,u_6,u_7,u_9,u_{13},u_{14}\}$<br>$\{u_5\} > \{u_2,u_{12}\}$
R_{38} $((((b,e),a),d),c)$	$\{u_{15}\} > \{u_3,u_{10}\}$<br>$\{u_5\} > \{u_2,u_{12}\} > \{u_4,u_6,u_7,u_9,u_{13},u_{14}\}$<br>$\{u_8\} > \{u_1,u_{11}\}$
R_{39} $((((b,e),c),a),d)$	$\{u_8\} > \{u_7,u_9\}$<br>$\{u_{15}\} > \{u_{13},u_{14}\} > \{u_1,u_3,u_4,u_6,u_{10},u_{11}\}$<br>$\{u_5\} > \{u_2,u_{12}\}$
R_{40} $((((b,e),c),d),a)$	$\{u_8\} > \{u_7,u_9\}$<br>$\{u_5\} > \{u_2,u_{12}\} > \{u_1,u_3,u_4,u_6,u_{10},u_{11}\}$<br>$\{u_{15}\} > \{u_{13},u_{14}\}$
R_{41} $((((b,e),d),a),c)$	$\{u_5\} > \{u_4,u_6\}$<br>$\{u_{15}\} > \{u_{13},u_{14}\} > \{u_2,u_3,u_7,u_9,u_{10},u_{12}\}$<br>$\{u_8\} > \{u_1,u_{11}\}$
R_{42} $((((b,e),d),c),a)$	$\{u_5\} > \{u_4,u_6\}$<br>$\{u_8\} > \{u_1,u_{11}\} > \{u_2,u_3,u_7,u_9,u_{10},u_{12}\}$<br>$\{u_{15}\} > \{u_{13},u_{14}\}$
R_{43} $((((c,d),a),b),e)$	$\{u_{15}\} > \{u_5,u_8\}$<br>$\{u_{10}\} > \{u_4,u_7\} > \{u_1,u_2,u_{11},u_{12},u_{13},u_{14}\}$<br>$\{u_3\} > \{u_6,u_9\}$
R_{44} $((((c,d),a),e),b)$	$\{u_{15}\} > \{u_5,u_8\}$<br>$\{u_3\} > \{u_6,u_9\} > \{u_1,u_2,u_{11},u_{12},u_{13},u_{14}\}$<br>$\{u_{10}\} > \{u_4,u_7\}$
R_{45} $((((c,d),b),a),e)$	$\{u_{10}\} > \{u_{11},u_{12}\}$<br>$\{u_{15}\} > \{u_{13},u_{14}\} > \{u_1,u_2,u_4,u_5,u_7,u_8\}$<br>$\{u_3\} > \{u_6,u_9\}$
R_{46} $((((c,d),b),e),a)$	$\{u_{10}\} > \{u_{11},u_{12}\}$<br>$\{u_3\} > \{u_6,u_9\} > \{u_1,u_2,u_4,u_5,u_7,u_8\}$<br>$\{u_{15}\} > \{u_{13},u_{14}\}$

Tree		Equivalence Classes
R_{47}	$((((c,d),e),a),b)$	$\{u_3\} > \{u_1,u_2\}$<br>$\{u_{15}\} > \{u_{13},u_{14}\} > \{u_5,u_6,u_8,u_9,u_{11},u_{12}\}$<br>$\{u_{10}\} > \{u_4,u_7\}$
R_{48}	$((((c,d),e),b),a)$	$\{u_3\} > \{u_1,u_2\}$<br>$\{u_{10}\} > \{u_4,u_7\} > \{u_5,u_6,u_8,u_9,u_{11},u_{12}\}$<br>$\{u_{15}\} > \{u_{13},u_{14}\}$
R_{49}	$((((c,e),a),b),d)$	$\{u_{14}\} > \{u_6,u_{11}\}$<br>$\{u_7\} > \{u_4,u_{10}\} > \{u_1,u_3,u_8,u_9,u_{13},u_{15}\}$<br>$\{u_2\} > \{u_5,u_{12}\}$
R_{50}	$((((c,e),a),d),b)$	$\{u_{14}\} > \{u_6,u_{11}\}$<br>$\{u_2\} > \{u_5,u_{12}\} > \{u_1,u_3,u_8,u_9,u_{13},u_{15}\}$<br>$\{u_7\} > \{u_4,u_{10}\}$
R_{51}	$((((c,e),b),a),d)$	$\{u_7\} > \{u_8,u_9\}$<br>$\{u_{14}\} > \{u_{13},u_{15}\} > \{u_1,u_3,u_4,u_6,u_{10},u_{11}\}$<br>$\{u_2\} > \{u_5,u_{12}\}$
R_{52}	$((((c,e),b),d),a)$	$\{u_7\} > \{u_8,u_9\}$<br>$\{u_2\} > \{u_5,u_{12}\} > \{u_1,u_3,u_4,u_6,u_{10},u_{11}\}$<br>$\{u_{14}\} > \{u_{13},u_{15}\}$
R_{53}	$((((c,e),d),a),b)$	$\{u_2\} > \{u_1,u_3\}$<br>$\{u_{14}\} > \{u_{13},u_{15}\} > \{u_5,u_6,u_8,u_9,u_{11},u_{12}\}$<br>$\{u_7\} > \{u_4,u_{10}\}$
R_{54}	$((((c,e),d),b),a)$	$\{u_2\} > \{u_1,u_3\}$<br>$\{u_7\} > \{u_4,u_{10}\} > \{u_5,u_6,u_8,u_9,u_{11},u_{12}\}$<br>$\{u_{14}\} > \{u_{13},u_{15}\}$
R_{55}	$((((d,e),a),b),c)$	$\{u_{13}\} > \{u_9,u_{12}\}$<br>$\{u_4\} > \{u_7,u_{10}\} > \{u_2,u_3,u_5,u_6,u_{14},u_{15}\}$<br>$\{u_1\} > \{u_8,u_{11}\}$
R_{56}	$((((d,e),a),c),b)$	$\{u_{13}\} > \{u_9,u_{12}\}$<br>$\{u_1\} > \{u_8,u_{11}\} > \{u_2,u_3,u_5,u_6,u_{14},u_{15}\}$<br>$\{u_4\} > \{u_7,u_{10}\}$
R_{57}	$((((d,e),b),a),c)$	$\{u_4\} > \{u_5,u_6\}$<br>$\{u_{13}\} > \{u_{14},u_{15}\} > \{u_2,u_3,u_7,u_9,u_{10},u_{12}\}$<br>$\{u_1\} > \{u_8,u_{11}\}$
R_{58}	$((((d,e),b),c),a)$	$\{u_4\} > \{u_5,u_6\}$<br>$\{u_1\} > \{u_8,u_{11}\} > \{u_2,u_3,u_7,u_9,u_{10},u_{12}\}$<br>$\{u_{13}\} > \{u_{14},u_{15}\}$
R_{59}	$((((d,e),c),a),b)$	$\{u_1\} > \{u_2,u_3\}$<br>$\{u_{13}\} > \{u_{14},u_{15}\} > \{u_5,u_6,u_8,u_9,u_{11},u_{12}\}$<br>$\{u_4\} > \{u_7,u_{10}\}$
R_{60}	$((((d,e),c),b),a)$	$\{u_1\} > \{u_2,u_3\}$<br>$\{u_4\} > \{u_7,u_{10}\} > \{u_5,u_6,u_8,u_9,u_{11},u_{12}\}$<br>$\{u_{13}\} > \{u_{14},u_{15}\}$

S2.2 Pseudo-caterpillar Trees

Table S3: Equivalence classes for pseudo-caterpillar 5-taxon rooted species trees. The u_is are named according to the unrooted trees in Table 5 in [1]. A script for generating these classes is available in the Quintet Rooting software repository in Github.

Tree		Equivalence Classes
R_{61}	$(((a, b), (c, d)), e)$	$\{u_3\} > \{u_1, u_2\}, \{u_{10}, u_{15}\}, \{u_6, u_9\} > \{u_4, u_5, u_7, u_8, u_{11}, u_{12}, u_{13}, u_{14}\}$
R_{62}	$(((a, c), (b, d)), e)$	$\{u_6\} > \{u_4, u_5\}, \{u_{11}, u_{14}\}, \{u_3, u_9\} > \{u_1, u_2, u_7, u_8, u_{10}, u_{12}, u_{13}, u_{15}\}$
R_{63}	$(((a, d), (b, c)), e)$	$\{u_9\} > \{u_7, u_8\}, \{u_{12}, u_{13}\}, \{u_3, u_6\} > \{u_1, u_2, u_4, u_5, u_{10}, u_{11}, u_{14}, u_{15}\}$
R_{64}	$(((a, b), (c, e)), d)$	$\{u_2\} > \{u_1, u_3\}, \{u_7, u_{14}\}, \{u_5, u_{12}\} > \{u_4, u_6, u_8, u_9, u_{10}, u_{11}, u_{13}, u_{15}\}$
R_{65}	$(((a, c), (b, e)), d)$	$\{u_5\} > \{u_4, u_6\}, \{u_8, u_{15}\}, \{u_2, u_{12}\} > \{u_1, u_3, u_7, u_9, u_{10}, u_{11}, u_{13}, u_{14}\}$
R_{66}	$(((a, e), (b, c)), d)$	$\{u_{12}\} > \{u_{10}, u_{11}\}, \{u_9, u_{13}\}, \{u_2, u_5\} > \{u_1, u_3, u_4, u_6, u_7, u_8, u_{14}, u_{15}\}$
R_{67}	$(((a, b), (d, e)), c)$	$\{u_1\} > \{u_2, u_3\}, \{u_4, u_{13}\}, \{u_8, u_{11}\} > \{u_5, u_6, u_7, u_9, u_{10}, u_{12}, u_{14}, u_{15}\}$
R_{68}	$(((a, d), (b, e)), c)$	$\{u_8\} > \{u_7, u_9\}, \{u_5, u_{15}\}, \{u_1, u_{11}\} > \{u_2, u_3, u_4, u_6, u_{10}, u_{12}, u_{13}, u_{14}\}$
R_{69}	$(((a, e), (b, d)), c)$	$\{u_{11}\} > \{u_{10}, u_{12}\}, \{u_6, u_{14}\}, \{u_1, u_8\} > \{u_2, u_3, u_4, u_5, u_7, u_9, u_{13}, u_{15}\}$
R_{70}	$(((a, c), (d, e)), b)$	$\{u_4\} > \{u_5, u_6\}, \{u_1, u_{13}\}, \{u_7, u_{10}\} > \{u_2, u_3, u_8, u_9, u_{11}, u_{12}, u_{14}, u_{15}\}$
R_{71}	$(((a, d), (c, e)), b)$	$\{u_7\} > \{u_8, u_9\}, \{u_2, u_{14}\}, \{u_4, u_{10}\} > \{u_1, u_3, u_5, u_6, u_{11}, u_{12}, u_{13}, u_{15}\}$
R_{72}	$(((a, e), (c, d)), b)$	$\{u_{10}\} > \{u_{11}, u_{12}\}, \{u_3, u_{15}\}, \{u_4, u_7\} > \{u_1, u_2, u_5, u_6, u_8, u_9, u_{13}, u_{14}\}$
R_{73}	$(((b, c), (d, e)), a)$	$\{u_{13}\} > \{u_9, u_{12}\}, \{u_1, u_4\}, \{u_{14}, u_{15}\} > \{u_2, u_3, u_5, u_6, u_7, u_8, u_{10}, u_{11}\}$
R_{74}	$(((b, d), (c, e)), a)$	$\{u_{14}\} > \{u_6, u_{11}\}, \{u_2, u_7\}, \{u_{13}, u_{15}\} > \{u_1, u_3, u_4, u_5, u_8, u_9, u_{10}, u_{12}\}$
R_{75}	$(((b, e), (c, d)), a)$	$\{u_{15}\} > \{u_5, u_8\}, \{u_3, u_{10}\}, \{u_{13}, u_{14}\} > \{u_1, u_2, u_4, u_6, u_7, u_9, u_{11}, u_{12}\}$

S2.3 Balanced Trees

Table S4: Equivalence classes for balanced 5-taxon rooted species trees. The u_is are named according to the unrooted trees in Table 5 in [1]. A script for generating these classes is available in the Quintet Rooting software repository in Github.

Tree		Equivalence Classes
R_{76}	$(((a,b),c),(d,e))$	$\{u_1\} > \{u_2,u_3\},\{u_4,u_{13}\} > \{u_5,u_6,u_9,u_{12}\} > \{u_7,u_8,u_{10},u_{11},u_{14},u_{15}\}$
R_{77}	$(((a,c),b),(d,e))$	$\{u_4\} > \{u_1,u_{13}\},\{u_5,u_6\} > \{u_2,u_3,u_9,u_{12}\} > \{u_7,u_8,u_{10},u_{11},u_{14},u_{15}\}$
R_{78}	$(((b,c),a),(d,e))$	$\{u_{13}\} > \{u_1,u_4\},\{u_9,u_{12}\} > \{u_2,u_3,u_5,u_6\} > \{u_7,u_8,u_{10},u_{11},u_{14},u_{15}\}$
R_{79}	$(((a,b),d),(c,e))$	$\{u_2\} > \{u_1,u_3\},\{u_7,u_{14}\} > \{u_6,u_8,u_9,u_{11}\} > \{u_4,u_5,u_{10},u_{12},u_{13},u_{15}\}$
R_{80}	$(((a,d),b),(c,e))$	$\{u_7\} > \{u_2,u_{14}\},\{u_8,u_9\} > \{u_1,u_3,u_6,u_{11}\} > \{u_4,u_5,u_{10},u_{12},u_{13},u_{15}\}$
R_{81}	$(((b,d),a),(c,e))$	$\{u_{14}\} > \{u_2,u_7\},\{u_6,u_{11}\} > \{u_1,u_3,u_8,u_9\} > \{u_4,u_5,u_{10},u_{12},u_{13},u_{15}\}$
R_{82}	$(((a,c),d),(b,e))$	$\{u_5\} > \{u_4,u_6\},\{u_8,u_{15}\} > \{u_3,u_7,u_9,u_{10}\} > \{u_1,u_2,u_{11},u_{12},u_{13},u_{14}\}$
R_{83}	$(((a,d),c),(b,e))$	$\{u_8\} > \{u_5,u_{15}\},\{u_7,u_9\} > \{u_3,u_4,u_6,u_{10}\} > \{u_1,u_2,u_{11},u_{12},u_{13},u_{14}\}$
R_{84}	$(((c,d),a),(b,e))$	$\{u_{15}\} > \{u_3,u_{10}\},\{u_5,u_8\} > \{u_4,u_6,u_7,u_9\} > \{u_1,u_2,u_{11},u_{12},u_{13},u_{14}\}$
R_{85}	$(((b,c),d),(a,e))$	$\{u_{12}\} > \{u_9,u_{13}\},\{u_{10},u_{11}\} > \{u_3,u_6,u_{14},u_{15}\} > \{u_1,u_2,u_4,u_5,u_7,u_8\}$
R_{86}	$(((b,d),c),(a,e))$	$\{u_{11}\} > \{u_6,u_{14}\},\{u_{10},u_{12}\} > \{u_3,u_9,u_{13},u_{15}\} > \{u_1,u_2,u_4,u_5,u_7,u_8\}$
R_{87}	$(((c,d),b),(a,e))$	$\{u_{10}\} > \{u_3,u_{15}\},\{u_{11},u_{12}\} > \{u_6,u_9,u_{13},u_{14}\} > \{u_1,u_2,u_4,u_5,u_7,u_8\}$
R_{88}	$(((a,b),e),(c,d))$	$\{u_3\} > \{u_1,u_2\},\{u_{10},u_{15}\} > \{u_5,u_8,u_{11},u_{12}\} > \{u_4,u_6,u_7,u_9,u_{13},u_{14}\}$
R_{89}	$(((a,e),b),(c,d))$	$\{u_{10}\} > \{u_3,u_{15}\},\{u_{11},u_{12}\} > \{u_1,u_2,u_5,u_8\} > \{u_4,u_6,u_7,u_9,u_{13},u_{14}\}$
R_{90}	$(((b,e),a),(c,d))$	$\{u_{15}\} > \{u_3,u_{10}\},\{u_5,u_8\} > \{u_1,u_2,u_{11},u_{12}\} > \{u_4,u_6,u_7,u_9,u_{13},u_{14}\}$
R_{91}	$(((a,c),e),(b,d))$	$\{u_6\} > \{u_4,u_5\},\{u_{11},u_{14}\} > \{u_2,u_7,u_{10},u_{12}\} > \{u_1,u_3,u_8,u_9,u_{13},u_{15}\}$
R_{92}	$(((a,e),c),(b,d))$	$\{u_{11}\} > \{u_6,u_{14}\},\{u_{10},u_{12}\} > \{u_2,u_4,u_5,u_7\} > \{u_1,u_3,u_8,u_9,u_{13},u_{15}\}$
R_{93}	$(((c,e),a),(b,d))$	$\{u_{14}\} > \{u_2,u_7\},\{u_6,u_{11}\} > \{u_4,u_5,u_{10},u_{12}\} > \{u_1,u_3,u_8,u_9,u_{13},u_{15}\}$
R_{94}	$(((b,c),e),(a,d))$	$\{u_9\} > \{u_7,u_8\},\{u_{12},u_{13}\} > \{u_2,u_5,u_{14},u_{15}\} > \{u_1,u_3,u_4,u_6,u_{10},u_{11}\}$
R_{95}	$(((b,e),c),(a,d))$	$\{u_8\} > \{u_5,u_{15}\},\{u_7,u_9\} > \{u_2,u_{12},u_{13},u_{14}\} > \{u_1,u_3,u_4,u_6,u_{10},u_{11}\}$
R_{96}	$(((c,e),b),(a,d))$	$\{u_7\} > \{u_2,u_{14}\},\{u_8,u_9\} > \{u_5,u_{12},u_{13},u_{15}\} > \{u_1,u_3,u_4,u_6,u_{10},u_{11}\}$
R_{97}	$(((a,d),e),(b,c))$	$\{u_9\} > \{u_7,u_8\},\{u_{12},u_{13}\} > \{u_1,u_4,u_{10},u_{11}\} > \{u_2,u_3,u_5,u_6,u_{14},u_{15}\}$
R_{98}	$(((a,e),d),(b,c))$	$\{u_{12}\} > \{u_9,u_{13}\},\{u_{10},u_{11}\} > \{u_1,u_4,u_7,u_8\} > \{u_2,u_3,u_5,u_6,u_{14},u_{15}\}$
R_{99}	$(((d,e),a),(b,c))$	$\{u_{13}\} > \{u_1,u_4\},\{u_9,u_{12}\} > \{u_7,u_8,u_{10},u_{11}\} > \{u_2,u_3,u_5,u_6,u_{14},u_{15}\}$
R_{100}	$(((b,d),e),(a,c))$	$\{u_6\} > \{u_4,u_5\},\{u_{11},u_{14}\} > \{u_1,u_8,u_{13},u_{15}\} > \{u_2,u_3,u_7,u_9,u_{10},u_{12}\}$
R_{101}	$(((b,e),d),(a,c))$	$\{u_5\} > \{u_4,u_6\},\{u_8,u_{15}\} > \{u_1,u_{11},u_{13},u_{14}\} > \{u_2,u_3,u_7,u_9,u_{10},u_{12}\}$
R_{102}	$(((d,e),b),(a,c))$	$\{u_4\} > \{u_1,u_{13}\},\{u_5,u_6\} > \{u_8,u_{11},u_{14},u_{15}\} > \{u_2,u_3,u_7,u_9,u_{10},u_{12}\}$
R_{103}	$(((c,d),e),(a,b))$	$\{u_3\} > \{u_1,u_2\},\{u_{10},u_{15}\} > \{u_4,u_7,u_{13},u_{14}\} > \{u_5,u_6,u_8,u_9,u_{11},u_{12}\}$
R_{104}	$(((c,e),d),(a,b))$	$\{u_2\} > \{u_1,u_3\},\{u_7,u_{14}\} > \{u_4,u_{10},u_{13},u_{15}\} > \{u_5,u_6,u_8,u_9,u_{11},u_{12}\}$
R_{105}	$(((d,e),c),(a,b))$	$\{u_1\} > \{u_2,u_3\},\{u_4,u_{13}\} > \{u_7,u_{10},u_{14},u_{15}\} > \{u_5,u_6,u_8,u_9,u_{11},u_{12}\}$

S3 Cost functions

We present the four cost functions studied in the paper, noting that the final cost function we selected was $Cost_4$. Each cost function is defined by its own way of weighting the different penalties for violating invariants or inequalities. $Cost_1$ only considers penalties for the invariants and not the inequalities, $Cost_2$ considers both but does not normalize them, and that $Cost_3$ and $Cost_4$ consider both types but use different weighting schemes.

$$Cost_1(R, \vec{\hat{u}}) = \underbrace{\sum_{c \in C_R} \frac{1}{|c|} \sum_{u_a, u_b \in c} |\hat{u}_a - \hat{u}_b|}_{\text{Invariants Penalty}} \tag{1}$$

$$Cost_2(R, \vec{\hat{u}}) = \underbrace{\sum_{c \in C_R} \sum_{u_a, u_b \in c} |\hat{u}_a - \hat{u}_b|}_{\text{Invariants Penalty}} + \underbrace{\sum_{c > c' \in C_R} \sum_{u_a \in c, u_b \in c'} max(0, \hat{u}_b - \hat{u}_a)}_{\text{Inequalities Penalty}} \tag{2}$$

$$Cost_3(R, \vec{\hat{u}}) = \underbrace{\sum_{c \in C_R} \frac{1}{|c|} \sum_{u_a, u_b \in c} |\hat{u}_a - \hat{u}_b|}_{\text{Invariants Penalty}} + \underbrace{\sum_{c > c' \in C_R} \frac{1}{|c|} \sum_{u_a \in c, u_b \in c'} max(0, \hat{u}_b - \hat{u}_a)}_{\text{Inequalities Penalty}} \tag{3}$$

$$Cost_4(R, \vec{\hat{u}}) = \underbrace{\sum_{c \in C_R} \frac{1}{|c|} \sum_{u_a, u_b \in c} |\hat{u}_a - \hat{u}_b|}_{\text{Invariants Penalty}} + \underbrace{\sum_{c > c' \in C_R} \frac{1}{|c'|} \sum_{u_a \in c, u_b \in c'} max(0, \hat{u}_b - \hat{u}_a)}_{\text{Inequalities Penalty}} \tag{4}$$

S4 Linear Encoding of Trees by Quintets

Since using all quintets for scoring different rootings can be computationally expensive for large trees, we propose a sparse sampling of quintets that leads to an optimized version of Quintet Rooting algorithm with overall complexity of $O(nk)$. Our experimental study in Section 4 (in the main paper) shows that this sparse sampling of quintets has a very similar accuracy to the original algorithm.

Encoding. Let R be a rooted binary tree with n leaves and let T denote its unrooted topology. For every edge e in $E(T)$, we define a quintet $q(e)$ of leaves in T so that e corresponds to a single edge in $q(e)$. The following cases can happen:

- (a) e is incident with a leaf x. In this case, e shares an endpoint with exactly two other edges, so these three edges together define a tripartition of the leafset of T into A, B, x, as shown in Figure S1. Form a quintet by picking x and at least one element from A and B, with the remaining two elements picked arbitrarily. Let $q(e)$ denote the tree induced on these five leaves by tree T.
- (b) e is not incident with any leaf, and so e shares an endpoint with exactly four other edges. These five edges together define a partition of the leafset of T into four sets A_1, A_2, B_1, and B_2, as shown in Figure S1. Pick one leaf from each of the four sets and the remaining leaf from any of the sets arbitrarily.

For each edge e we have defined a quintet of leaves, and we will denote by $q(e)$ the tree induced on these five leaves by tree T. Note that the set of trees in these quintets $Q^* = \{q(e) : q \in E(T)\}$ uniquely defines the tree T. Note also that if each $q(e)$ is rooted correctly, then the rooted tree R can be inferred.

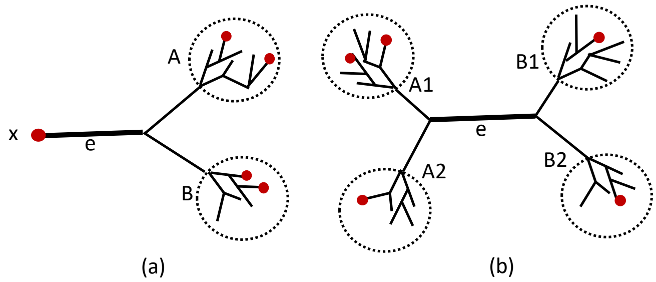

Figure S1: Linear mapping of edges in a tree to quintets of taxa. (a) edge e is adjacent to a leaf or (b) edge e shares an endpoint with four other edges.

The number of edges in an unrooted binary tree with n leaves is $2n-3$, and therefore this encoding uses $O(n)$ quintets. Hence, the overall runtime of the Quintet Rooting algorithm with this sampling becomes $O(nk)$.

S5 Relationship between Root Distance and Clade Distance

Let R be a rooted binary tree with n leaves. For every node v, let R_v denote the subtree of tree R below node v and $\mathcal{L}(R_v)$ denote the leafset of subtree R_v. The set of clades of R is defined as $Clades(R) = \{\mathcal{L}(R_v) : v \in V(R)\}$.

For two binary trees R and R' with n leaves and the same unrooted topology T, we define the *root distance* of R and R', denoted by $RD(R, R')$, as the number of nodes on the path between the root nodes of these trees. We also define the *clade distance* of R and R', denoted by $CD(R, R')$, as the symmetric difference between $Clades(R)$ and $Clades(R')$; thus $CD(R, R') =$

$$|Clades(R) \triangle Clades(R')| = |Clades(R) \backslash Clades(R')| + |Clades(R') \backslash Clades(R)| \quad (5)$$

We now prove the following lemma:

Lemma 1. *For rooted binary trees R and R' with unrooted topology T, we have $CD(R, R') = 2RD(R, R')$.*

Proof. Let r denote the root node of R and r' denote the root of R'. Since R and R' have the same unrooted topology T, let e and e' be edges of the tree T containing the roots r and r' (a rooted tree can be created by picking up the unrooted topology at any edge). Let $P = \{v_1, v_2, \ldots, v_k\}$ be the set of nodes on the path between e and e' in T; note that $|P| = k$ is the root distance between R and R'. For every vertex $v \in V(T)$, we can consider the clades in R and R' that are rooted at v, and the clade distance between R and R' is twice the number of all nodes that define different clades. However, it is easy to see that any node $v \notin P$ defines a clade that is in both R and R', and also every node $v \in P$ defines different clades in R and R'. Hence, the clade distance between R and R' is $2|P|$, so that $CD(R, R') = 2|P| = 2 \times RD(R, R')$. □

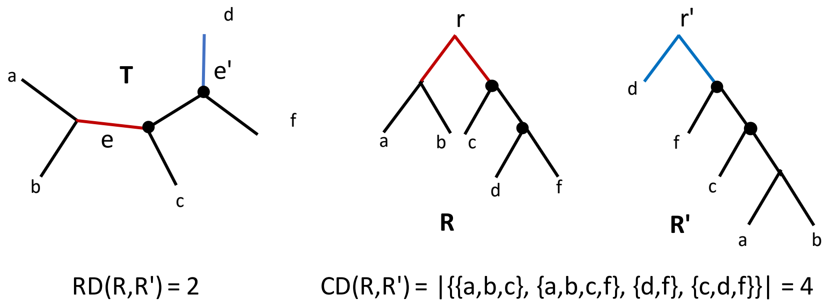

Figure S2: Relationship between clade distance and root distance for an example 5-taxon tree T, with different rootings R and R'. Only nodes on path P between r and r' define different clades, and are counted twice in the clade distance between two trees.

S6 Additional Results

S6.1 Rooting larger trees

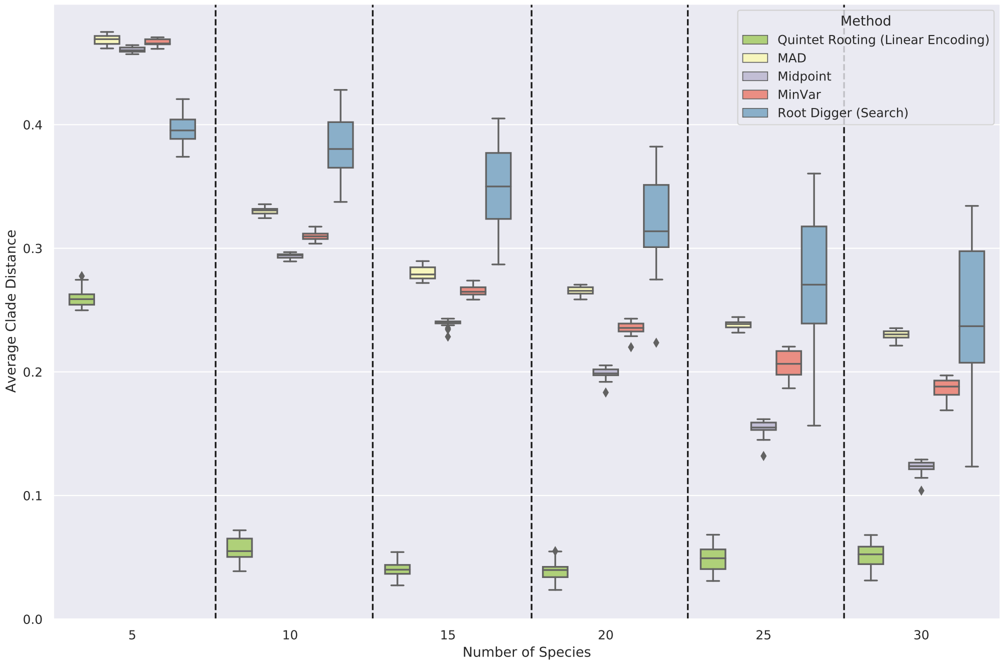

Figure S3: Average clade distance on subsets of the avian simulated datasets with 5 to 30 leaves for each rooting method, given the true species tree topology and true gene trees. The branch lengths on the model tree are estimated using RAxML with the concatenated gene multiple sequence alignments. The results are averaged over 200 samples for each value of k. The number of genes is 1000 and the error bars are shown across 20 replicates.

S6.2 Rooting Accuracy

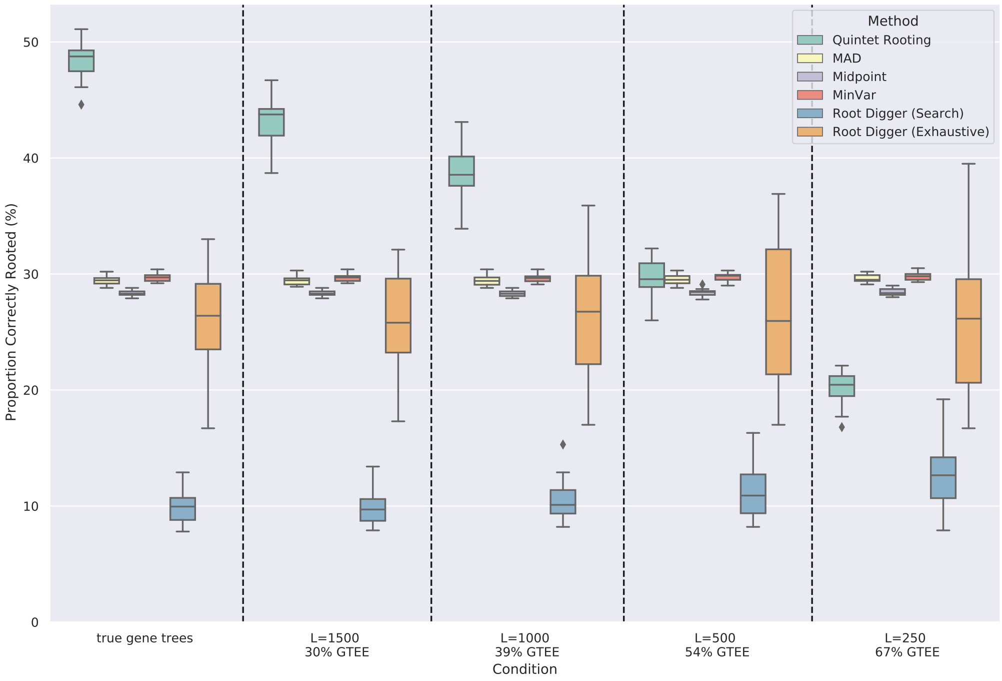

Figure S4: Proportion of the 5-leaf subtrees of the avian model tree correctly rooted by each rooting method. The results are averaged over 1000 sample 5-species trees. The number of genes is 1000 and the error bars are shown across 20 replicates. All methods root the true species tree topology and branch lengths are estimated using RAxML on the concatenated gene sequence alignments.

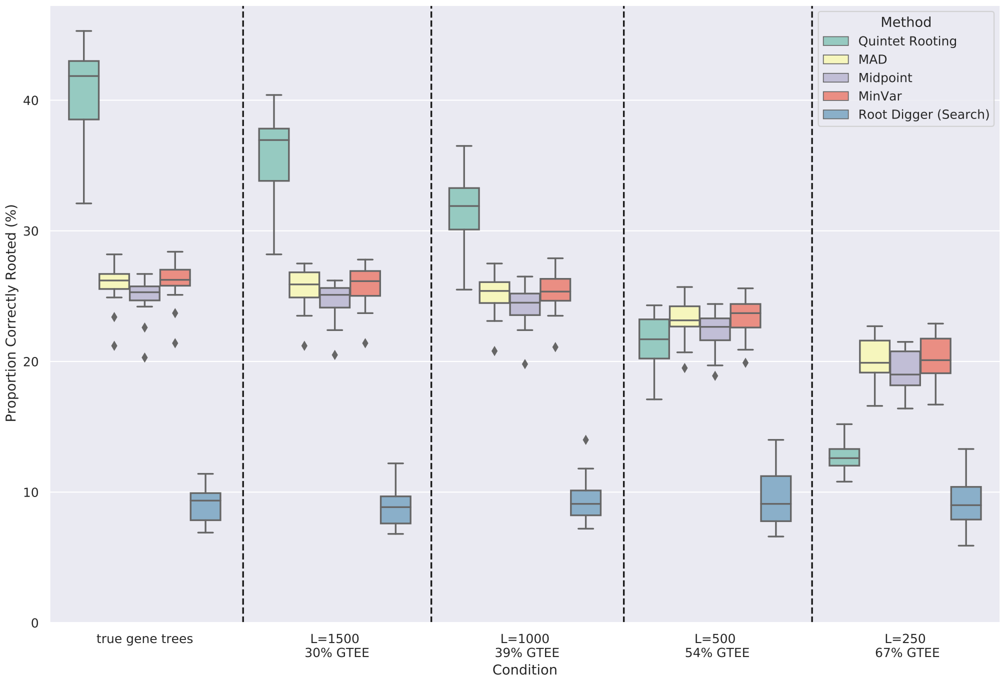

Figure S5: Proportion of the trees correctly rooted on 5-leaf avian simulated datasets by each rooting method given an estimated species tree computed by ASTRAL. The branch lengths on the estimated species tree are estimated using RAxML with the concatenated gene multiple sequence alignments. The results are averaged over 1000 sample 5-species trees. The number of genes is 1000 and the error bars are shown across 20 replicates.

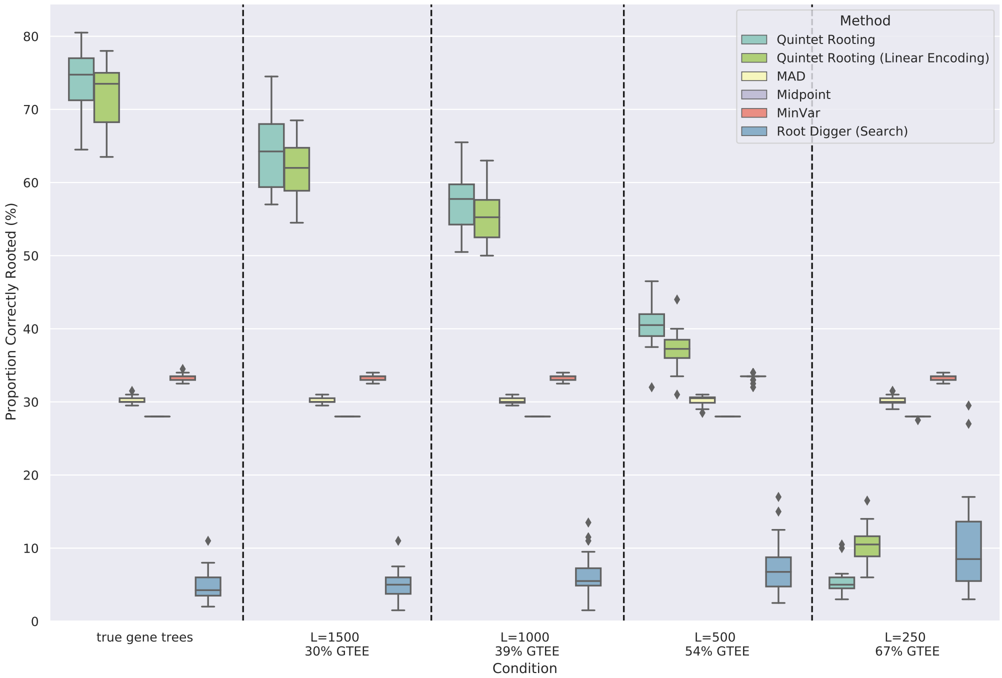

Figure S6: Proportion of the trees correctly rooted on 10-leaf avian simulated datasets given the true (model) species tree by each rooting method. The results are averaged over 200 sample 10-species trees. The number of genes is 1000 and the error bars are shown across 20 replicates. The branch lengths are estimated using RAxML on the concatenated gene sequence alignments.

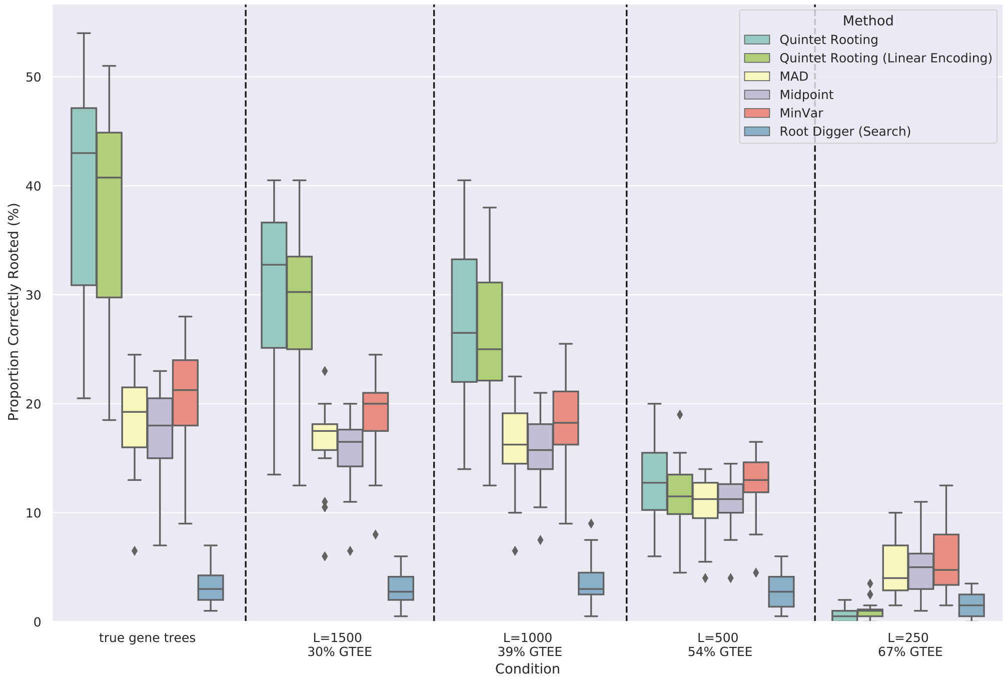

Figure S7: Proportion of the trees correctly rooted on 10-leaf avian simulated datasets by each rooting method given an estimated species tree computed by ASTRAL. The branch lengths on the ASTRAL tree are estimated using RAxML with the concatenated gene multiple sequence alignments. The results are averaged over 200 sample 10-species trees. The number of genes is 1000 and the error bars are shown across 20 replicates.

References

[1] Elizabeth S. Allman, James H. Degnan, and John A. Rhodes. "Identifying the rooted species tree from the distribution of unrooted gene trees under the coalescent". In: *J. Math. Biol.* 62 (2011), pp. 833–862.

[2] Jaime Huerta-Cepas, François Serra, and Peer Bork. "ETE 3: Reconstruction, Analysis, and Visualization of Phylogenomic Data". In: *Mol. Biol. Evol.* 33.6 (2016), pp. 1635–1638.

[3] Erich D Jarvis, Siavash Mirarab, Andre J Aberer, Bo Li, Peter Houde, Cai Li, Simon YW Ho, Brant C Faircloth, Benoit Nabholz, Jason T Howard, et al. "Phylogenomic analyses data of the avian phylogenomics project". In: *GigaScience* 4.1 (2015), s13742–014.

[4] Erich D. Jarvis, Siavash Mirarab, and et al. "Whole-genome analyses resolve early branches in the tree of life of modern birds". In: *Science* 346.6215 (2014), pp. 1320–1331.

[5] Jeet Sukumaran and Mark T Holder. "DendroPy: a Python library for phylogenetic computing". In: *Bioinformatics* 26.12 (2010), pp. 1569–1571.
